# The Benzothiazine Core as a Novel Motif for DNA-Binding Small Molecules

**DOI:** 10.3390/molecules28114499

**Published:** 2023-06-01

**Authors:** Milena Mlakić, Ivona Čipor, Petra Kovačec, Goran Kragol, Ana Ratković, Tatjana Kovačević, Rahela Zadravec, Valentina Milašinović, Krešimir Molčanov, Ivo Piantanida, Irena Škorić

**Affiliations:** 1 Department of Organic Chemistry, Faculty of Chemical Engineering and Technology, University of Zagreb, Marulićev trg 19, HR-10000 Zagreb, Croatia; mdragojev@fkit.hr (M.M.); pkovacec@fkit.hr (P.K.); 2 Division of Organic Chemistry and Biochemistry, Ruđer Bošković Institute, HR-10000 Zagreb, Croatia; icipor@irb.hr; 3 Chemistry, Selvita Ltd., Prilaz Baruna Filipovića 29, HR-10000 Zagreb, Croatia; goran.kragol@selvita.com (G.K.); ana.ratkovic@selvita.com (A.R.); tatjana.kovacevic@selvita.com (T.K.); rahela.zadravec@selvita.com (R.Z.); 4 Division of Physical Chemistry, Rudjer Bošković Institute, Bijenička Cesta 54, HR-10000 Zagreb, Croatia; valentina.milasinovic@irb.hr (V.M.); kresimir.molcanov@irb.hr (K.M.)

**Keywords:** 4*H*-1,3-benzothiazines, electrochemical dehydrogenative cyclization, DNA/RNA binding, UV/vis spectroscopy, circular dichroism

## Abstract

A new series of 4*H*-1,3-benzothiazine dyes were prepared and fully characterized in an aqueous medium. Benzothiazine salts were synthesized either through the classical synthetic pathway using Buchwald–Hartwig amination or through economical and environmentally friendly electrochemical synthesis. The latest synthetic approach employs successful electrochemical intramolecular dehydrogenative cyclization of *N*-benzylbenzenecarbothioamides to form 4*H*-1,3-benzothiazines. 4*H*-1,3-Benzothiazines were evaluated as novel DNA/RNA probes. Through the use of several methods such as UV/vis spectrophotometric titrations, circular dichroism and thermal melting experiments, the binding of four benzothiazine-based molecules to polynucleotides was examined. Compounds **1** and **2** acted as DNA/RNA groove binders, thus suggesting the potential of these compounds as novel DNA/RNA probes. This is a proof-of-concept study and will be expanded to include SAR/QSAR studies.

## 1. Introduction

Benzothiazines are a promising scaffold in medicinal chemistry, with numerous biological activities [1]. The benzothiazine moiety can be found in some commercially available drugs, such as the oxicam family of nonsteroidal anti-inflammatory drugs [2]. Various benzothiazines have been shown to possess anti-inflammatory [3], antimicrobial [4] and antiproliferative [5] properties, which makes them an attractive motif to investigate in the context of medicinal chemistry and chemical biology.

Studying the noncovalent interactions of small molecules that target biomolecules such as DNA and RNA is an important point of research in biomedical sciences [6]. Such molecules bind to ds-DNA/RNA through one or sometimes several dominant binding modes, such as intercalation between base pairs, groove binding or electrostatic interactions between the polycationic small molecule and the strongly negatively charged DNA/RNA backbone [7]. The type of dominant noncovalent interaction is strongly related to the structure of the small molecule as well as to the secondary structure of ds-DNA or ds-RNA [8]. The information gathered through these kinds of investigations can be extremely useful for either drug design and optimization or the development of novel DNA/RNA probes. While benzothiazole-derived small molecules are commonly explored in medicinal chemistry [9] and investigated for their DNA-binding abilities [10,11], benzothiazine is a less researched scaffold, especially in the context of DNA binding despite the preferable photochemical properties of some of its derivatives. However, some benzothiazine derivatives have already been employed as sensors [12,13]. For instance, 1,4-benzothiazine hydrazide was employed as a colorimetric and fluorimetric reversible chemosensor for mercury ions [14]. Frequently, the benzothiazine unit is part of a larger ring-fused system, such as Δ^2,2′^-bi-(2*H*-1,4-benzothiazine), which occurs in trichochrome pigments found in red hair and feathers [15]. This nature-inspired chromophore has been fashioned into a cyanine-type dye and employed as a colorimetric pH sensor [16]. Various transition metal complexes of oxicam drugs have been synthesized, characterized and assessed as DNA-binding small molecules [17,18], but there are no systematic studies on benzothiazine substituents controlling the DNA-binding affinity and spectroscopic response, which prompted us to prepare and investigate the novel benzothiazine compounds.

In this work, four benzothiazine-based small molecule dyes were synthesized using both conventional and green chemistry methods, and their binding affinities for DNA/RNA were determined using spectroscopic methods such as UV/vis spectroscopy and circular dichroism.

## 2. Results and Discussion

### 2.1. Synthesis and Spectroscopic Characterization of the New Benzothiazine Salts ***1***–***4***

**Scheme 1:**
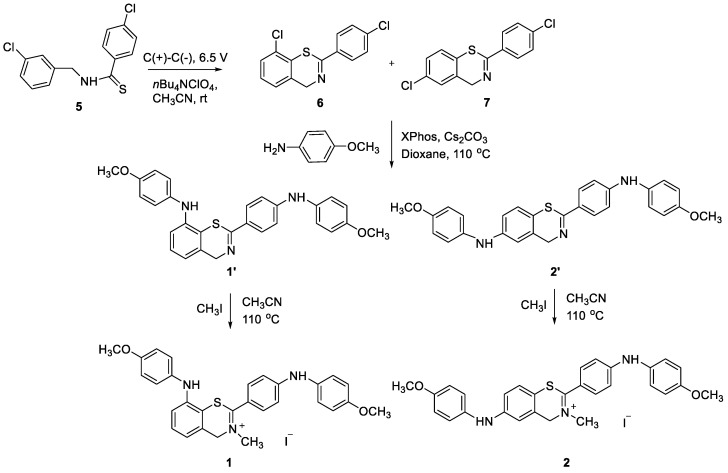
Reaction pathway for the synthesis of benzothiazines **1** and **2**.

Benzothiazine salts **1** and **2** were synthesized in several steps (Scheme 1) starting from the thioamide **5** and its electrochemical ring closure to the mixture of 4*H*-1,3-benzothiazines **6** and **7** [19,20]. The mixture was stirred and electrolyzed (constant voltage = 6.5 V) for 30 min at room temperature under air using an electrochemical reactor and carbon plates as the cathode and anode. Five identical reactions were performed and combined for the work up. The residue was purified using flash chromatography to afford a mixture of 4*H*-1,3-benzothiazines **6** and **7** in a ~1:1 ratio, according to HPLC and NMR analyses. In the second step, the amination reaction of the mixture of **6** and **7** in dioxane using 4-methoxyaniline, Cs_2_CO_3_ and XPhos Pd G3 was carried out. The crude residue was purified using flash column chromatography on silica gel to afford a mixture of 1,3-benzothiazines **1′** and **2′**, separated using preparative HPLC (eluent 0.1% of formic acid in water/acetonitrile) to afford **1′** (26%) and **2′** (28%), which are substrates for the methylation reaction giving benzothiazine salts **1** and **2** (Scheme 1; see also Section 3 and ESI).

**Scheme 2:**
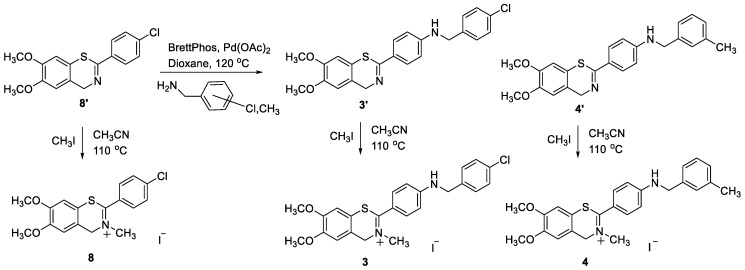
Reaction pathway for the synthesis of benzothiazines **3**, **4** and **8**.

Compounds **3′** and **4′** (Scheme 2) were synthesized using the Buchwald–Hartwig amination of chloro-substituted benzothiazine **8′** using BrettPhos, Pd(OAc)_2_ in dioxane, water, corresponding benzylamine and KO*t*Bu in a sealed tube. The reaction mixtures were purified through repeated column chromatography using a PE/DCM (20–50%) mixture as the eluent. The obtained amines **3′** and **4′** were isolated in the first fractions as orange oils.

The methylation reaction was first performed on the test compound **8′** to convert it into **8** (Scheme 2) to establish the reaction conditions and conversions for the benzothiazine structures **1′**–**4′**; only after that, we proceeded to the methylation of amines **1′**–**4′** to **1**–**4** (Scheme 2; see also Section 3). Before the addition of the reagents, the reaction solutions for methylations were purged with nitrogen for 10 min. Methylations were carried out by adding three equivalents of methyl iodide to a solution of the neutral amines **1′**–**4′** in acetonitrile. The reactions were quenched using evaporating solvents, obtaining the crude products **1**–**4**, which were worked up using column chromatography and detected in the last fractions.

**Figure 1:**
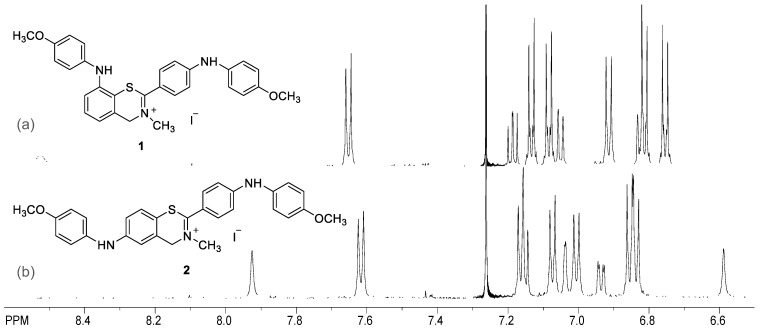
Aromatic parts of the ^1^H NMR spectra (CDCl_3_) of benzothiazines **1** (**a**) and **2** (**b**).

The obtained benzothiazine salts **1**–**4** were isolated in moderate to high yields (30–50%) and completely characterized using NMR spectroscopy and HRMS analyses (Figure 1; see also Section 3 and ESI).

**Figure 2:**
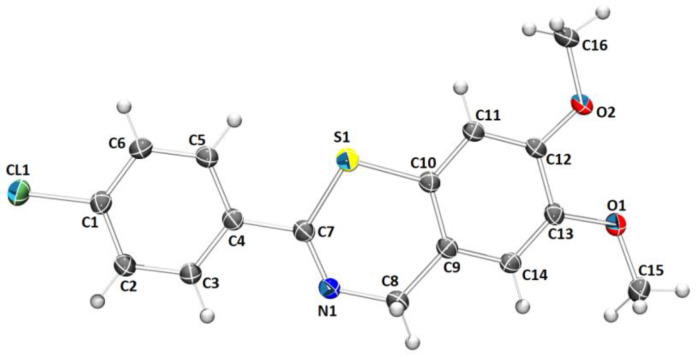
Molecular structure of compound **8′**. Displacement ellipsoids are drawn for the probability of 20% and hydrogen atoms are shown as spheres of arbitrary radii.

The molecular structure of the test compound **8′** was determined and is shown in Figure 2. The compound crystallized in the space group *P*2_1_/*n*, with the molecular symmetry C*_2h_*.

**Figure 3:**
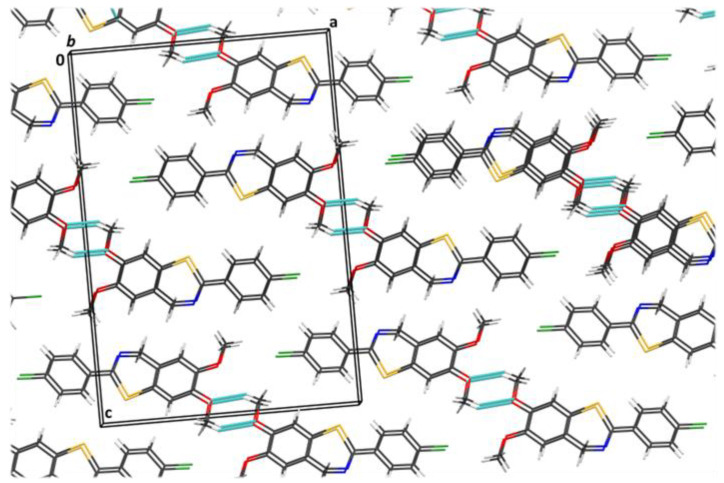
Crystal packing of **8′** is characterized by hydrogen bonds and stacking interactions.

In the crystal packing of **8′**, the intermolecular hydrogen bond (C16–H16C⋯O_2_) links two molecules into dimers (Figure 3 and Table 1 and Table 2), which stacks in the direction [010]. There is also one intramolecular hydrogen bond, C5–H5⋯S1.

### 2.2. Spectroscopic Characterization and Interactions with Biomolecules

To examine the spectroscopic properties of the newly synthetized compounds, UV/vis spectroscopy was employed. Further, several polynucleotides were selected to analyze the interactions with biomolecules due to the significantly different secondary structure of their homogenous polynucleotide sequences. Specifically, in this study, the experiments were performed with naturally isolated DNA from calf thymus (ct-DNA), which is characterized by an equal amount of AT- and GC- base pairs and a typical B-helical structure; poly A–poly U as a representative of the typical RNA A-helical structure, which contains a major groove as a potential target for small molecules, and poly dAdT–poly dAdT, characterized by a B-helical structure and a minor groove that can bind small molecules [8].

#### 2.2.1. UV/Vis Spectra and Photophysical Characterization

**Figure 4:**
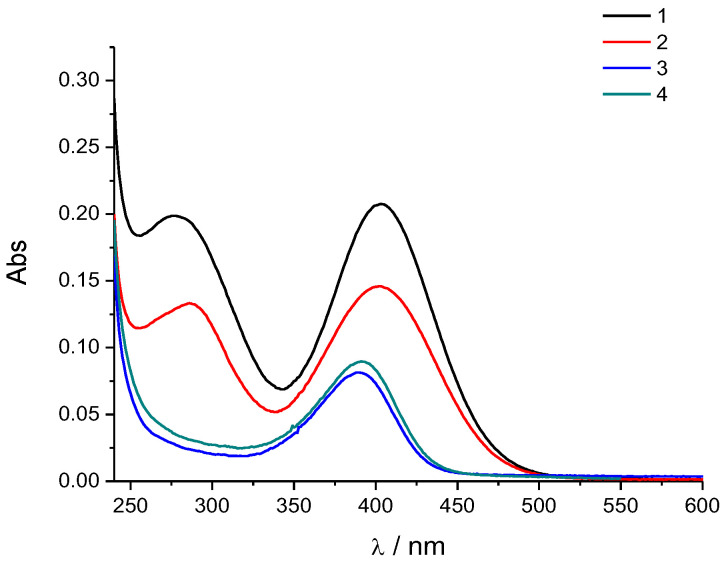
UV/vis spectra of studied compounds (*c* = 1 × 10^−5^ M) in water.

All the compounds were moderately soluble in water; however, for ease of handling, we prepared their stock solutions in DMSO (0.001 M). When stored at 4 °C, these stock solutions were stable for a long period. The absorbancies of the aqueous solutions of all the compounds were proportional to their concentrations up to *c* = 2 × 10^−5^ M (ESI, Figure S1). Upon temperature increase, the UV/vis spectra of all compounds (Figure 4) showed negligible changes (ESI, Figure S2). Thus, the compounds did not show aggregation in water under the given experimental conditions. Generally, the amino-aryl-functionalized 1,3-benzothiazines **1** and **2** showed two absorption maximums and higher values of the molar extinction coefficient, along with red-shifted wavelengths of maximum absorbance in contrast to the chloro- or methoxy-substituted compounds **3** and **4** (Figure 1 and Table 1). The position of the amino-aryl moiety on the 1,3-benzothiazine core also plays a significant role in the electronic absorption properties (Table 3); hence, compound **1**, with the substituent at C8 of the ring showed a larger molar absorptivity in contrast to its C6-substituted analog **2**. The aqueous solutions of compounds did not emit fluorescence under the given experimental conditions.

#### 2.2.2. UV/Vis Spectrophotometric Titrations

**Figure 5:**
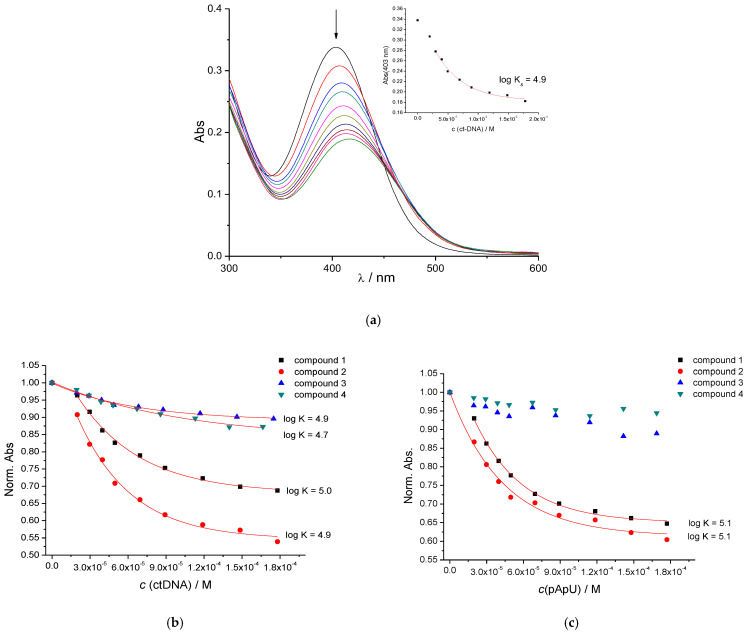
(**a**) Changes in the UV/vis spectra of **2** (*c* = 2 × 10^−5^ M) upon titration with ct-DNA, inset: absorbance changes at λ_max_ = 403 nm, fitted to Scatchard eq. (red line) [21]; (**b**) normalized absorbance changes at the respective λ_max_ for all studied compounds upon titration with ct-DNA, fitted to Scatchard eq. (red line) [21]; (**c**) normalized absorbance changes at the respective λ_max_ for all studied compounds upon titration with pApU, fitted to Scatchard eq. (red line) [21].

Upon the addition of ct-DNA, the UV/vis spectra of **1**–**4** showed pronounced hypochromic and bathochromic shifts (Figure 5a,b; ESI, Figure S3). However, only the UV/vis spectra of **1** and **2** changed significantly upon the addition of ds-RNA (Figure 5c), whereas the UV/vis spectra of **3** and **4** showed only negligible changes (ESI, Figure S5). The addition of AT-DNA to compounds **1** and **2** also resulted in strong bathochromic and hypochromic shifts as well as the appearance of very similar isosbestic points (ESI, Figure S4). For most of the studied compounds, the isosbestic points in titrations with ct-DNA and AT-DNA (Figure 5a; ESI, Figure S3) suggest the formation of one dominant type of compound/polynucleotide complex and allowing the calculation of the binding constants by fitting the experimental data to the McGhee–von Hippel formalism of the Scatchard equation [21] (Table 4). The similar values of the binding constants obtained for AT-DNA and ctDNA (48% of GC base pairs) suggest that compounds **1**, **2** bind to similar binding sites with a comparable set of noncovalent interactions. However, in experiments with RNA, a systematic deviation from the isosbestic point suggested the formation of several different binding sites of which one is likely dominant (ESI, Figure S5); therefore, fitting to the Scatchard equation could only give an estimate of the cumulative binding constant for all the binding sites included.

#### 2.2.3. CD Experiments

To further investigate the binding of the compounds to DNA/RNA, circular dichroism spectroscopy was utilized. This spectroscopic method is sensitive to changes in the secondary structure of the chiral DNA/RNA helix, which can occur upon the binding of small molecules to polynucleotides [22]. Further, achiral small molecules can obtain an induced circular dichroism spectrum (ICD) when binding to chiral polynucleotides, whereby the sign, shape and strength of the ICD band can suggest a dominant mode of interaction if corroborated by other methods [23].

**Figure 6:**
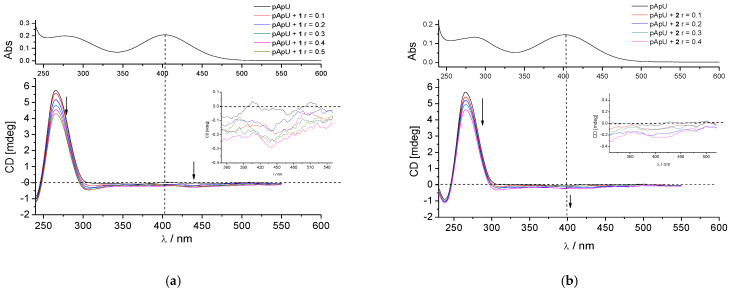
CD titration of pApU (c = 2 × 10^−5^ M) with **1** and **2** at molar ratios r = [compound]/[polynucleotide] (pH 7.0, buffer sodium cacodylate, I = 0.05 M). (**a**) pApU with **1**, inset: ICD at 440 nm, (**b**) pApU with **2**, inset: ICD at 410 nm.

The addition of **1** or **2** yielded changes in the CD spectrum of ds-DNA or ds-RNA (260–300 nm region; ESI, Figures S6, S7, S10 and S11), suggesting the unwinding of the double helix of polynucleotide [22,23]. The addition of **3** and **4** resulted in less pronounced, almost negligible, changes in the 260–300 nm range. Moreover, in some titrations (Figure 6; ESI, Figures S8, S10 and S11), weak, negative induced CD bands appeared, commonly associated with the intercalative binding mode [24]. However, some groove binders could also yield such ICD bands; therefore, an additional judicative method is necessary to confirm the binding mode.

#### 2.2.4. Thermal Melting Experiments

The thermal denaturation of ds-DNA/RNA occurs at a specific temperature (*T_m_* value). This process can, therefore, be used in the investigation of the helical secondary structure of DNA/RNA or in the characterization of noncovalent complexes formed by the binding of small molecules to polynucleotides [24]. An increase in the *T_m_* value signifies the stabilization of the ds-polynucleotide, whereas a decrease points to the destabilization of the polynucleotide. The difference between the *T_m_* of the free polynucleotide and the *T_m_* of the polynucleotide/small molecule complex can be used to determine the binding mode. For example, moderate to strong stabilization (Δ*T_m_* > 5 °C) supports intercalation or, in the case of polycationic small molecules, eventual minor-groove binding [25], while weak to moderate stabilization (Δ*T_m_* = 0–5 °C) suggests a binding process driven by electrostatic interactions, the hydrophobic effect, weak H-bonding or a combination of the mentioned interactions.

The addition of dyes **1**–**4** to ct-DNA or AT-DNA at a ratio r = 0.3 resulted in a small decrease in the *T_m_* value (Table 5; ESI, Figures S14–S17, S22 and S23), whereas the addition of these compounds to ds-RNA resulted in negligible stabilization (Table 5; ESI, Figures S17–S21); the only exception being weak stabilization for +3.5 °C observed for the **2**/RNA complex. Such mostly negligible effects do not support the intercalative binding mode but are more in line with hydrophobic-driven binding within polynucleotide grooves.

## 3. Materials and Methods

### 3.1. General Remarks

All the used solvents were commercially available and were purified by distillation. Firstly, the purity of all products was checked using thin-layer chromatography on silica gel (0.2 mm, Kiselgel 60 F_254_), and spots were detected using a UV lamp. Column chromatography was performed on columns using silica gel (60 Å, technical grade). The silica gel was packed as a petroleum ether suspension. Nuclear magnetic resonance (NMR) spectroscopic data from ^1^H and ^13^C NMR were recorded at room temperature on the spectrometers Bruker Avance (Bruker, New York, NY, USA) 300 and 600 MHz (ESI, Figures S24–S52). Deuterated chloroform, CDCl_3_, with tetramethylsilane and deuterated dimethyl sulfoxide, DMSO-*d*_6_, as standards were used for recording the NMR spectra. The chemical shifts were reported in parts per million. The abbreviations used in this experimental procedure are as follows: NMR—nuclear magnetic resonance, UV—ultraviolet spectrophotometry, PE—petroleum ether, DCM—dichloromethane, MeOH—methanol, CH_3_CN—acetonitrile, EtOAc—ethyl-acetate. All solvents were removed from the solutions using a rotary evaporator under reduced pressure. All compounds were dissolved in DMSO at 1 × 10^−3^ M. The stock solutions were kept at +4 °C and showed no visible precipitation. Working aliquots were prepared from the stock solutions before measurements and kept at 25 °C. Polynucleotides were purchased as noted: *calf thymus* (ct)–DNA (Aldrich) and poly dAdT–poly dAdT (AT-DNA in text) and poly A–poly U(pApU in text) (Sigma) and dissolved in sodium cacodylate buffer, *I* = 0.05 M, pH = 7.0. The ct-DNA was additionally sonicated and filtered through a 0.45 mm filter to obtain mostly short (ca. 100 base pairs) rod-like B-helical DNA fragments [26]. The polynucleotide concentration was determined spectroscopically [23] as the concentration of phosphates, which corresponds to *c*(nucleobase).

### 3.2. Spectrophotometric Studies and Interaction with DNA or RNA

UV/vis spectra were obtained on a Varian Cary 100 Bio spectrometer (SpectraLab Scientific Inc., Markham, ON, Canada), and UV/vis titrations were performed on an Agilent Cary 60 spectrometer (SpectraLab Scientific Inc., Markham, ON, Canada). The circular dichroism spectra were recorded on a JASCO J815 spectropolarimeter (Equip, San Diego, CA, USA). The experiments were performed in 1 cm pathlength quartz cuvettes.

The UV/vis titrations were performed in aqueous buffer solutions (sodium cacodylate buffer, pH = 7, I = 0.05 M), by adding aliquots of ds-polynucleotide stock solutions to solutions of the compounds and monitoring the changes in the spectra of compounds in the range > 300 nm, to avoid the absorbance of the DNA/RNA. Changes in the UV/vis spectrum were processed using a nonlinear fit to the McGhee–von Hippel formalism of the Scatchard equation [21], to calculate the binding constants.

The circular dichroism titrations were performed in a sodium cacodylate buffer (pH = 7, I = 0.05 M) by adding portions of the compound stock solution into a buffered solution of ds-polynucleotide.

Thermal melting experiments were performed in a sodium cacodylate buffer (pH = 7, I = 0.05 M) by adding the compound stock solution to ds-DNA/RNA (c = 2 × 10^−5^ M) in ratios of r = [compound]/[polynucleotide] = 0.2 or 0.3. The thermal denaturation curves were obtained by monitoring the absorption change at 260 nm as a function of temperature [24]. The *T_m_* values were determined as the midpoints of the denaturation curve determined from the maximum of the first derivative and checked using the tangent method [27]. The Δ*T_m_* values were obtained as the difference of the *T_m_* values for DNA/RNA–compound complexes and the *T_m_* values for free polynucleotides. Every reported Δ*T_m_* value is an average of at least two measurements, and the error of the method is ±0.5 °C.

### 3.3. Electrochemical Ring Closure for Benzothiazine Core Synthesis

To a solution of 20 mg (0.068 mmol) of the thioamide **5** in acetonitrile (20 mL), 11.5 mg (0.034 mmol) of *n*Bu_4_NClO_4_ was added. The mixture was stirred and electrolyzed (constant voltage = 6.5 V) for 30 min at room temperature under air using an IKA ElectraSyn 2.0 reactor (IKA^®^-Werke GmbH & Co., Staufen, Germany) and carbon plates (2.64 cm^2^) as the cathode and anode. Five identical reactions were performed and combined for the work up. The solvent was evaporated, and the residue was dissolved in ethyl acetate (100 mL) followed by washing with a saturated aqueous solution of NaHCO_3_ (1 × 30 mL) and a saturated aqueous solution of NaCl (2 × 30 mL). The organic layer was dried over anhydrous Na_2_SO_4_, filtered and evaporated. The residue was purified using flash chromatography on silica gel by gradient elution (0–10% of ethyl acetate in cyclohexane) to afford 50 mg of a mixture of 4*H*-1,3-benzothiazines **6** and **7** in a ~1:1 ratio.

### 3.4. Amination Reaction of 4H-1,3-benzothiazines ***6*** and ***7***

**Scheme 3:**
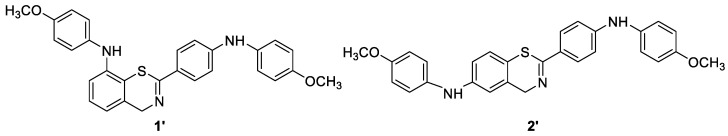
Structure of compounds **1′** and **2′**.

To a solution of 50.0 mg (0.170 mmol) of a mixture of 4*H*-1,3-benzothiazines **6** and **7** in dioxane (5 mL), 62.8 mg (0.51 mmol) of 4-methoxyaniline and 221.5 mg (0.68 mmol) of Cs_2_CO_3_ were added. The mixture was purged with nitrogen for 5 min, and then 32 mg of XPhos Pd G3 (0.068 mmol) was added. The mixture was shaken in a sealed tube at 110 °C for 2 h. After the removal of the solvent, the crude residue was purified using flash column chromatography on silica gel by gradient elution (0–10% of DCM:MeOH: NH_4_OH (90:9:0.5) in DCM) to afford a mixture of 1,3-benzothiazines **1′** and **2′**. The obtained mixture was then separated using preparative HPLC (Instrument Agilent 1260 Infinity II (LC/MSD), column SunFire C18 19 × 100 (5 µm), flow 30 mL/min, 6 min run, eluent 0.1% of formic acid in water/acetonitrile) to afford 13.5 mg of **1′** and 14.9 mg of **2′** (Scheme 3).

*N*-(4-methoxyphenyl)-2-(4-((4-methoxyphenyl)amino)phenyl)-4*H*-benzo[*e*][1,3]thiazin-8-amine (**1′**): 13 mg (isolated 26%); orange oil; R*_f_* (DCM/MeOH = 9/1) = 0.85; ^1^H NMR (CDCl_3_, 600 MHz) *δ*/ppm: 7.94 (d, *J* = 8.4 Hz, 2H), 7.13–7.17 (m, 5H), 6.97 (d, *J* = 7.8 Hz, 1H), 6.88–6.93 (m, 7H), 5.80 (s, 1H), 5.75 (s, 1H), 4.76 (s, 2H), 3.84 (s, 6H); ^13^C NMR (CDCl_3_, 150 MHz) *δ*/ppm: 55.5, 57.4, 112.6, 114.0, 114.7 (2 × CH), 117.4, 118.1, 123.4, 123.7, 127.4, 127.6, 129.5, 133.5, 134.0, 134.9, 142.1, 148.2, 155.9, 156.1, 160.2; ^1^H NMR (DMSO-d_6_, 600 MHz) *δ*/ppm: 8.35 (s, 1H, NH), 7.82 (d, *J* = 8.9 Hz, 2H), 7.39 (s, 1H, NH), 7.18 (t, *J* = 7.8 Hz, 1H), 7.11 (d, *J* = 8.8 Hz, 2H), 7.00 (d, *J* = 8.8 Hz, 2H), 6.97 (d, *J* = 7.8 Hz, 1H), 6.95 (d, *J* = 7.8 Hz, 1H), 6.91 (dd, *J* = 8.8; 1.5 Hz, 4H), 6.87 (d, *J* = 8.9 Hz, 2H), 4.62 (s, 2H), 3.73 (s, 3H), 3.72 (s, 3H); HRMS (*m*/*z*) for C_28_H_25_N_3_O_2_S: [M + H]^+^_calcd_ = 467.1668; [M + H]^+^_measured_ = 467.1662.

*N*-(4-methoxyphenyl)-2-(4-((4-methoxyphenyl)amino)phenyl)-4*H*-benzo[*e*][1,3]thiazin-6-amine (**2′**): 14 mg (isolated 28%); orange oil; R*_f_* (DCM/MeOH = 9/1) = 0.81; ^1^H NMR (CDCl_3_, 600 MHz) *δ*/ppm: 7.88 (d, *J* = 8.4 Hz, 2H), 7.21 (d, *J* = 8.4 Hz, 1H), 7.15 (d, *J* = 8.4 Hz, 2H), 7.08 (d, *J* = 8.4 Hz, 2H), 6.84–6.93 (m, 8H), 5.80 (s, 1H), 5.58 (s, 1H), 4.67 (s, 2H), 3.84 (s, 3H), 3.83 (s, 3H); ^13^C NMR (CDCl_3_, 150 MHz) *δ*/ppm: 55.5, 56.7, 113.9, 114.0, 114.7 (2 × CH), 115.1, 120.5, 122.4, 123.6, 127.3, 127.8, 129.3, 133.4, 134.1, 135.3, 145.0, 148.1, 155.5, 156.0, 162.3; ^1^H NMR (DMSO-d_6_, 600 MHz) *δ*/ppm: 8.32 (s, 1H, NH), 7.96 (s, 1H, NH), 7.75 (d, *J* = 8.9 Hz, 2H), 7.20 (d, *J* = 8.7 Hz, 1H), 7.10 (d, *J* = 8.7 Hz, 2H), 7.05 (d, *J* = 8.9 Hz, 2H), 6.95 (d, *J* = 2.7 Hz, 1H), 6.92–6.89 (m, 4H), 6.88–6.86 (m, 3H), 4.55 (s, 2H), 3.72 (s, 3H), 3.71 (s, 3H); HRMS (*m*/*z*) for C_28_H_25_N_3_O_2_S: [M + H]^+^_calcd_ = 467.1668; [M + H]^+^_measured_ = 467.1663.

### 3.5. Synthesis of Compounds ***3′*** and ***4′***

**Scheme 4:**
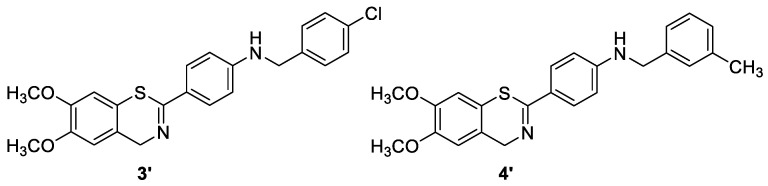
Structure of compounds **3′** and **4′**.

Compounds **3′** and **4′** were synthesized using the Buchwald–Hartwig amination of chloro-substituted benzothiazine **8′**. To a solution of 0.1 equivalent of BrettPhos and 0.05 equivalent of Pd(OAc)_2_ in dioxane, 4% of water was added. After the addition of water, the solution was heated to 120 °C for 2 min. The reaction mixture changed color from light yellow over dark red to dark green. Benzothiazine **8′**, corresponding benzylamine (2 equivalent), and KO*t*Bu (1.4 equivalent) were added into a tube, which was sealed. The reaction mixtures were stirred for 20 h at 180 °C. After the removal of the solvent, the crude residue was purified through repeated column chromatography using a PE/DCM (20–50%) mixture as the eluent. The obtained amines **3′** and **4′** were isolated in the first fractions as yellow oils, while BrettPhos remained on the chromatographic column (Scheme 4).

*N*-[(4-chlorophenyl)methyl]-4-(6,7-dimethoxy-4*H*-1,3-benzothiazin-2-yl)aniline (**3′**): 23 mg (isolated 32%); orange oil; *R*_f_ (EtOAc) = 0.64; ^1^H NMR (CDCl_3_, 600 MHz) *δ*/ppm: 7.89 (d, *J* = 8.2 Hz, 2H), 7.27–7.33 (m, 6H), 6.88 (s, 1H), 6.85 (s, 1H), 6.62 (d, *J* = 8.5 Hz, 1H), 4.69 (s, 3H), 4.38 (s, 3H); ^13^C NMR (CDCl_3_, 150 MHz) *δ*/ppm: 142.7, 141.1, 129.5, 125.5, 121.9, 121.3, 121.2, 121.0, 120.9, 116.6, 114.7, 104.6, 104.5, 102.4, 101.9, 48.5, 48.4, 39.5; HRMS (*m*/*z*) for C_23_H_21_ClN_2_O_2_S: [M + H]^+^_calcd_ = 424.1012, [M + H]^+^_measured_ = 424.1004.

4-(6,7-Dimethoxy-4*H*-1,3-benzothiazin-2-yl)-*N*-(*m*-tolylmethyl)aniline (**4′**): 24 mg (isolated 37%); orange oil; *R*_f_ (EtOAc) = 0.72; ^1^H NMR (DMSO-d_6_, 600 MHz) *δ*/ppm: 2.27 (s, 3H), 3.75 (s, 3H), 3.76 (s, 3H), 4.27 (d, *J* = 5.9 Hz, 2H), 4.56 (s, 2H), 6.58–6.64 (m, 2H), 6.85 (t, *J* = 6.0 Hz, 1H), 6.98 (s, 1H), 7.02–7.07 (m, 2H), 7.10–7.22 (m, 3H), 7.67 (m, *J* = 8.8 Hz, 2H); ^13^C NMR (DMSO-d_6_, 150 MHz) *δ*/ppm: 159.4, 151.4, 148.4, 148.3, 139.5, 137.4, 128.6, 128.2, 127.7, 127.4, 124.3, 123.9, 123.8, 121.2, 111.6, 110.7, 109.9, 55.8, 55.7, 55.0, 45.9, 21.0; HRMS (*m*/*z*) for C_24_H_24_N_2_O_2_S: [M + H]^+^_calcd_ = 404.1453; [M + H]^+^_measured_ = 404.1447.

### 3.6. Methylation of Amines ***1′***–***4′*** to the Final Salts ***1***–***4*** and Test Compound ***8′*** to ***8***

**Scheme 5:**
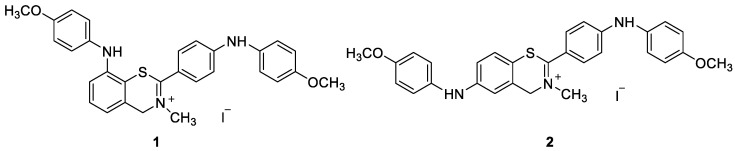
Structure of compounds **1** and **2**.

The final salts **1**–**4** were synthesized through the methylation of amines **1′**–**4′**. The reaction solutions were purged with nitrogen for 10 min before adding the reagents. Methylations were carried out by adding 3 equivalents of methyl iodide to a solution of the neutral amines **1′**–**4′** in acetonitrile. The mixtures were stirred for 120 h (5 days) at 80 °C. The reactions were quenched using evaporating solvents, leaving the crude products **1**–**4**, which were worked up through column chromatography using a DCM/MeOH (5%) mixture as the eluent and detected in the last fractions (Scheme 5).

8-((4-Methoxyphenyl)amino)-2-(4-((4-methoxyphenyl)amino)phenyl)-3-methyl-4*H*-benzo[*e*][1,3]thiazin-3-ium iodide (**1**): 5 mg (isolated 50%); red oil; R*_f_* (DCM/MeOH = 9/1) = 0.41; ^1^H NMR (CDCl_3_, 600 MHz) *δ*/ppm: 7.65 (d, *J* = 8.9 Hz, 2H), 7.19 (t, *J* = 7.8 Hz, 1H), 7.13 (d, *J* = 8.8 Hz, 2H), 7.08 (d, *J* = 8.8 Hz, 2H), 7.05 (d, *J* = 8.5 Hz, 1H), 6.91 (d, *J* = 8.8 Hz, 2H), 6.83–6.79 (m, 3H), 6.75 (d, *J* = 8.8 Hz, 2H), 4.82 (s, 2H), 3.76 (s, 3H), 3.74 (s, 3H), 3.73 (s, 3H) (2 signals for NH protons are missing); ^13^C NMR (CDCl_3_, 600 MHz) *δ*/ppm: 178.2, 156.8, 155.8, 153.4, 143.3, 134.9, 134.6, 132.0, 131.8, 130.6, 124.3, 123.0, 118.3, 116.7, 115.5, 114.8, 114.6, 113.9, 60.5, 55.6, 55.5, 46.7; HRMS (*m*/*z*) for C_29_H_27_N_3_O_2_S: [M + H]^+^_calcd_ = 481.1824; [M + H]^+^_measured_ = 481.1827.

**Scheme 6:**
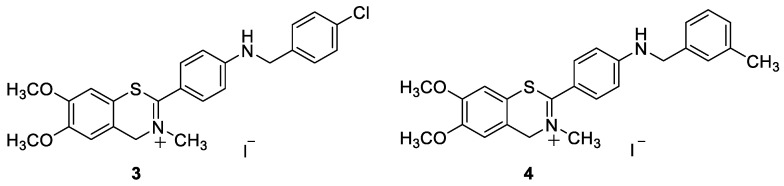
Structure of compounds **3** and **4**.

6-((4-Methoxyphenyl)amino)-2-(4-((4-methoxyphenyl)amino)phenyl)-3-methyl-4*H*-benzo[*e*][1,3]thiazin-3-ium iodide (**2**): 6 mg (isolated 45%); red oil; R*_f_* (DCM/MeOH = 9/1) = 0.39; ^1^H NMR (CDCl_3_, 600 MHz) *δ*/ppm: 7.93 (s, 1H, NH), 7.62 (d, *J* = 8.5 Hz, 2H), 7.18–7.13 (m, 3H), 7.07 (d, *J* = 8.6 Hz, 2H), 7.04 (s, 1H), 7.01 (d, *J* = 8.5 Hz, 2H), 6.93 (d, *J* = 8.4 Hz, 1H), 6.87–6.82 (m, 4H), 6.59 (s, 1H, NH), 4.88 (s, 2H), 3.78 (s, 6H), 3.77 (s, 3H); ^13^C NMR (CDCl_3_, 600 MHz) *δ*/ppm: 179.5, 157.2, 156.2, 153.4, 148.4, 134.0, 133.6, 131.9, 131.8, 126.4, 124.9, 123.9, 116.0, 114.8, 114.7, 114.5, 114.2, 114.0, 113.6, 59.9, 55.6, 55.5, 47.0; HRMS (*m*/*z*) for C_29_H_27_N_3_O_2_S: [M + H]^+^_calcd_ = 481.1824; [M + H]^+^_measured_ = 481.1820. (Scheme 6)

2-(4-((4-Chlorobenzyl)amino)phenyl)-6,7-dimethoxy-3-methyl-4*H*-benzo[*e*][1,3]thiazin-3-ium iodide (**3**): 7 mg (isolated yield 30%); orange-red oil; R*_f_* (EtOAc) = 0.21; ^1^H NMR (CDCl_3_ + DMSO-d_6_, 600 MHz) *δ*/ppm: 8.01 (d, *J* = 8.0 Hz, 2H); 7.65 (d, *J* = 8.8 Hz, 2H), 7.40–7.34 (m, 3H), 7.17 (s, 1H), 6.92 (s, 1H), 6.72 (d, *J* = 8.4 Hz, 2H), 4.99 (s, 2H), 4.38 (d, *J* = 5.8 Hz, 2H), 3.92 (s, 3H), 3.89 (s, 3H), 3.86 (s, 3H); ^13^C NMR (DMSO-d_6_, 600 MHz) *δ*/ppm: The compound disintegrated upon standing in DMSO, and there was not enough left to record this technique. HRMS (*m*/*z*) for C_24_H_23_ClN_2_O_2_S: [M + H]^+^_calcd_ = 438.1169; [M + H]^+^_measured_ = 438.1165.

**Scheme 7:**
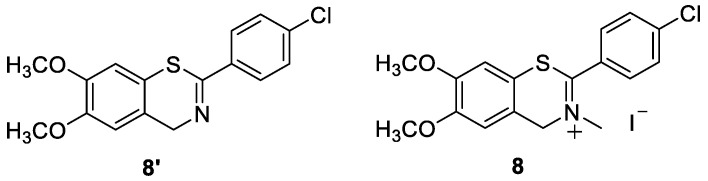
Structure of compounds **8′** and **8**.

6,7-Dimethoxy-3-methyl-2-(4-(3-methylphenethyl)phenyl)-4*H*-benzo[*e*][1,3]thiazin-3-ium iodide (**4**): 10 mg (isolated yield 41%); orange-red oil; R*_f_* (EtOAc) = 0.16; ^1^H NMR (CDCl_3_+ DMSO-d_6_, 600 MHz) *δ*/ppm: 7.70 (d, *J* = 8.9 Hz, 2H), 7.37 (t, *J* = 7.9 Hz, 1H), 7.21–7.16 (m, 2H), 7.10–7.03 (m, 3H), 6.91 (s, 1H), 6.74 (d, *J* = 7.9 Hz, 2H), 6.11 (t, *J* = 5.8 Hz, 1H, NH), 5.04 (s, 2H), 4.39 (d, *J* = 5.8 Hz, 2H), 3.93 (s, 3H), 3.92 (s, 3H), 3.86 (s, 3H); ^13^C NMR (DMSO-d_6_, 600 MHz) *δ*/ppm: The compound disintegrated upon standing in DMSO, and there was not enough left to record this technique. HRMS (*m*/*z*) for C_25_H_26_N_2_O_2_S: [M + H]^+^_calcd_ = 418.1715; [M + H]^+^_measured_ = 418.1713 (Scheme 7).

2-(4-Chlorophenyl)-6,7-dimethoxy-4*H*-benzo[*e*][1,3]thiazine (**8′**): bright-yellow powder; m.p. 137–138 °C; R*_f_* (DCM/MeOH = 9/1) = 0.65; ^1^H NMR (CDCl_3_, 600 MHz) *δ*/ppm: 7.93 (d, *J* = 9.1 Hz, 2H), 7.39 (d, *J* = 8.1 Hz, 2H), 6.85 (d, *J* = 6.8 Hz, 2H), 4.72 (s, 2H), 3.89 (d, *J* = 3.5 Hz, 6H); HRMS (*m*/*z*) for C_16_H_14_ClNO_2_S: [M + H]^+^_calcd_ = 319.0434; [M + H]^+^_measured_ = 319.0432.

2-(4-Chlorophenyl)-6,7-dimethoxy-3-methyl-4*H*-benzo[*e*][1,3]thiazin-3-ium iodide (**8**): 35 mg (isolated yield70%); yellow powder; m.p. 205–206 °C; R*_f_* (DCM/MeOH = 9/1) = 0.18; ^1^H NMR (CDCl_3_, 600 MHz) *δ*/ppm: 8.09 (d, *J* = 8.9 Hz, 1H), 7.63 (d, *J* = 8.9 Hz, 1H), 7.36 (s, 2H), 6.91 (s, 1H), 5.56 (s, 1H), 4.04 (s, 2H), 3.93 (s, 3H), 3.98 (s, 3H), 3.92 (s, 3H), 3.89 (s, 3H); ^13^C NMR (CDCl_3_, 600 MHz) *δ*/ppm: 151.6, 150.5, 142.4, 132.4, 130.4, 128.9, 128.5, 119.4, 115.9, 111.2, 107.7, 60.8, 56.4, 48.3; HRMS (*m*/*z*) for C_17_H_16_ClNO_2_S: [M + H]^+^_calcd_ = 333.0590; [M + H]^+^_measured_ = 333.0582.

### 3.7. X-ray Crystallography

Single-crystal measurements were performed on an XtaLAB Synergy diffractometer, using micro-focus sealed X-ray tube Cu*K_α_* (1.54184 Å) radiation at room temperature (293(2) K). The CrysAlisPRO package (Rigaku OD, 2018) was used for data reduction and numerical absorption correction. The structure was solved with SHELXS97 [28] and refined with SHELXL2018 [29]. The model was refined using the full-matrix least-squares refinement; all nonhydrogen atoms were refined anisotropically. The hydrogen atoms were located in a difference Fourier map and refined as a mixture of free and riding entities. The molecular geometry calculations were performed with PLATON, [30] and the molecular graphics were prepared using ORTEP-3 [31] and CCDC-Mercury [32]. The crystallographic and structure-refinement data for the structure reported in this paper are shown in Table 6. The supplementary crystallographic data for this paper can be obtained free of charge via www.ccdc.cam.ac.uk/conts/retrieving.html (accessed on 15 April 2023) (or from the Cambridge Crystallographic Data Centre, 12, Union Road, Cambridge CB2 1EZ, UK; fax: +44 1223 336033; or deposit@ccdc.cam.ac.uk). CCDC-2235600 and CCDC-2235603 contain supplementary crystallographic data for this paper.

## 4. Conclusions

A new series of benzothiazine dyes **1**–**4** were prepared and fully characterized in an aqueous medium. Derivatives **1** and **2**, characterized by an amino-aryl substituent at the benzothiazine core, showed red-shifted absorption maxima in comparison to the methoxy-substituted **3** and **4**. In addition, **1** and **2** showed moderate (10 µM) affinity toward ds-DNA and ds-RNA, accompanied by moderate changes in their UV/vis spectra and characterized by a dominant single binding mode (binding to DNA/RNA grooves), at variance to the low affinity and mixed binding modes of **3** and **4**. The obtained results make **1** and **2** more promising dyes for biorelevant applications, particularly taking into account their innovative chemical structure (benzothiazine), and support their further optimization in line with the design of their fluorescent analogs. This is a proof-of-concept study and will be expanded to include SAR/QSAR studies.

## Figures and Tables

**Table 1:** Geometric parameters of π interactions for compound **8′**.

*π*⋯*π*	Cg ^a^⋯Cg/Å	*α* ^b^/º	*β* ^c^/º	Cg⋯Plane(Cg2)/Å	Offset/Å	Symm.
C1→C6⋯C1→C6	4.1579(15)	0	32.1	3.5235(10)	2.208	*x*, −1 + *y*, z
C9→C14⋯C9→C14	4.1580(12)	0.02(10)	27.9	3.6733(9)	1.949	*x*, −1 + *y*, z

^a^ Cg = center of gravity of the ring; ^b^ *α* = angle between planes of two interacting rings; ^c^ *β* = angle between the Cg⋯Cg line and the normal to the plane of the first interacting ring.

**Table 2:** Geometric parameters of hydrogen bonds determined from geometric analysis for compound **8′**.

	*D*–H/Å	H⋯*A*/Å	*D*⋯*A*/Å	*D*–H⋯*A*/º	Symm. op. on *A*
C5–H5⋯S1	0.93	2.65	3.054(2)	107	*x*, *y*, *z*
C16–H16C⋯O_2_	0.96	2.70	3.543(2)	146	−*x*, −*y*, −*z*

**Table 3:** Electronic absorption properties of examined compounds in water.

Compound	λ_max_/nm	ε/M^−1^ cm^−1^
**1**	278	19,977
	402	20,982
**2**	286	13,455
	402	14,780
**3**	388	7956
**4**	392	9030

**Table 4:** Binding constant (^a^ logKs) values calculated from UV/vis titrations with ds-polynucleotides, at pH = 7.0, I = 0.05 M, in sodium cacodylate buffer.

Compound	ct-DNA	pApU	AT-DNA
**1**	5.0	^c^ 5.1	4.9
**2**	4.9	^c^ 5.1	4.9
**3**	4.9	^b^	-
**4**	4.7	^b^	-

^a^ Processing of the titration data using the Scatchard equation [21] gave values of the ratio of n[bound dye]/[polynucleotide] = 0.2 and 0.3. For ease of comparison, all logK*_s_* values were recalculated for fixed *n* = 0.2. The correlation coefficients were >0.99 for all calculated K*_s_*. ^b^ Too small changes for accurate processing using the Scatchard eq. [21]. ^c^ Estimate of the cumulative binding constant for all the binding modes included.

**Table 5:** Δ*T_m_* values for different ratios ^a^*r* of tested compounds added to polynucleotides obtained from thermal dissociation experiments.

Compound	*r*	ctDNA	AT-DNA	pApU
**1**	0.2	-	−1.8 ± 0.5 °C	+2 ± 0.5 °C
	0.3	−2 ± 0.5 °C	−2.1 ± 0.5 °C	−1.2 ± 0.5 °C
**2**	0.2	−2.3 ± 0.5 °C	-	-
	0.3	−1± 0.5 °C	−1.5 ± 0.5 °C	+3.5 ± 0.5 °C
**3**	0.3	-	-	+1 ± 0.5 °C
**4**	0.3	−1.5 ± 0.5 °C	-	−1.5 ± 0.5 °C

^a^*r* = [compound]/[polynucleotide].

**Table 6:** Crystallographic data and structure refinement details for compound **8′**.

Compound	8′
Empirical formula	C_16_H_14_ClNO_2_S
Formula wt./g mol_^−1^_	319.79
Crystal dimensions/mm	0.45 × 0.3 × 0.2
Space group	*P*2_1_/*n*
*a*/Å	15.6901 (4)
*b*/Å	4.15810 (10)
*c/*Å	22.5946 (5)
*α*/°	90
*β*/°	90.042 (2)
*γ*/°	90
*Z*	4
*V*/Å^3^	1474.09 (6)
*D*_calc_/g cm^−3^	1.441
*μ*/mm^−1^	3.647
*Θ* range/°	3.428–80.123
*T*(K)	293 (2)
Radiation wavelength	1.54184 (Cu*K*_*α*_)
Diffractometer type	XtaLAB Synergy, Dualflex, HyPix
Range of *h*, *k*, *l*	−20 > *h* > 19; −5 > *k* > 5; −27 > *l* > 28
Reflections collected	18295
Independent reflections	3186
Observed reflections (*I* ≥ 2σ)	2895
*R_int_*	0.0542
*R (F)*	0.0523
*R_w_ (F^2^)*	0.1537
No. of parameters, restraints	192, 0
Goodness of fit	1.085
Δ*ρ*_max_, Δ*ρ*_min_ (eÅ^−3^)	0.355; −0.299

## Data Availability

The data presented in this study are available on request from the corresponding author. The data are not publicly available due to privacy.

## Supplementary Materials

The following supporting information can be downloaded at https://www.mdpi.com/article/10.3390/molecules28114499/s1: Physico-chemical properties of solutions (Figures S1 and S2); Interactions with ds-DNA/RNA (Figures S3–S23); 1H and 13C NMR spectra of compounds **1**–**4** and **1′**–**4′**, **8** and **8′** (Figures S24–S42); MS and HRMS analyses of compounds **1**–**4** and **1′**–**4′**, **8** and **8′** (Figures S43–S52).

## Sample Availability

Not available.
