# Supplementary material for: The Benzothiazine Core as a Novel Motif for DNA-Binding Small Molecules
Source: Molecules. 2023 Jun 1;28(11):4499. doi: 10.3390/molecules28114499 (PMC10254314; doi:10.3390/molecules28114499)
Supplement: Supplementary file 1 [file molecules-28-04499-s001.zip › molecules-2398652-supplementary.pdf]

Supplementary material

The benzothiazine core as a novel motif for DNA-binding small molecules

Milena Mlakić [1,†], Ivona Čipor [2,†], Petra Kovačec [1,3], Goran Kragol [3], Ana Ratković [3], Tatjana Kovačević [3], Rahela Zadravec [3], Valentina Milašinović [4], Krešimir Molčanov [4], Ivo Piantanida [2,*] and Irena Škorić [1,*]

1 Department of Organic Chemistry, Faculty of Chemical Engineering and Technology, University of Zagreb, Marulićev trg 19, HR-10000 Zagreb, Croatia; mdragojev@fkit.hr (M.M.); pkovacec@fkit.hr (P.K.)

2 Division of Organic Chemistry and Biochemistry, Ruđer Bošković Institute, icipor@irb.hr (I.Č.)

3 Chemistry, Selvita Ltd., Prilaz Baruna Filipovića 29, HR-10000 Zagreb, Croatia; goran.kragol@selvita.com (G.K.); ana.ratkovic@selvita.com (A.R.); tatjana.kovacevic@selvita.com (T.K.); rahela.zadravec@selvita.com (R.Z.)

4 Division of Physical Chemistry, Rudjer Bošković Institute, Bijenička cesta 54, HR-10000 Zagreb, Croatia; valentina.milasinovic@irb.hr (V.M.); kresimir.molcanov@irb.hr (K.M.)

Contents

1. Physico-chemical properties of aqueous solutions 2

2. Interactions with ds-DNA/RNA 5

3. NMR spectra of benzothiazines 16

4. MS spectra and HRMS analyses of benzothiazines 26

1. Physico-chemical properties of aqueous solutions

All compounds were dissolved in DMSO at 1 × 10^{-3} M. The stock solutions were kept at +4 °C and working aliquots were kept at +25 °C. No visible precipitation in working solutions was noticed. The experiments were performed in MQ water at room temperature (25 °C).

1.1. UV/Vis spectra

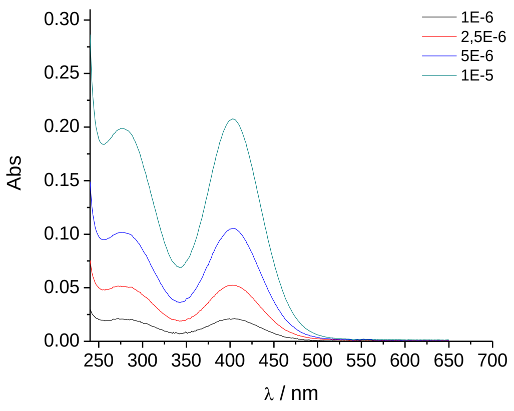

a)

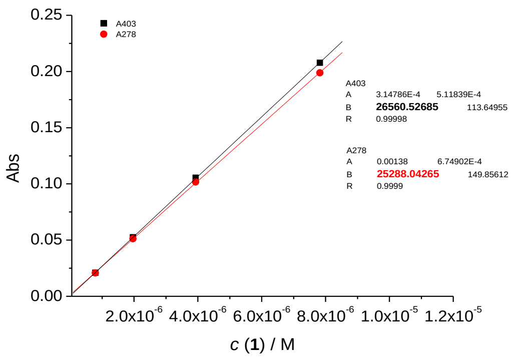

b)

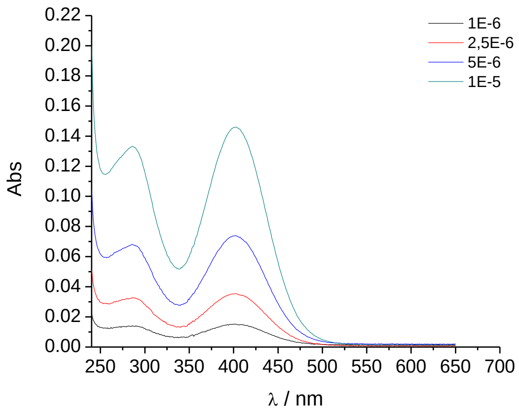

c)

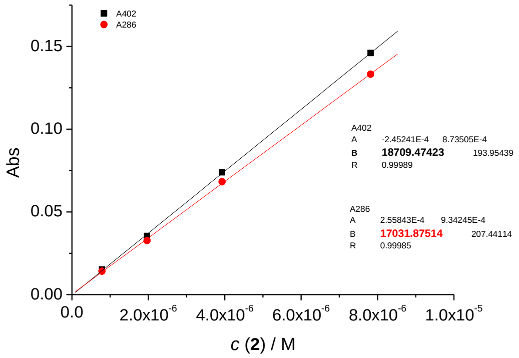

d)

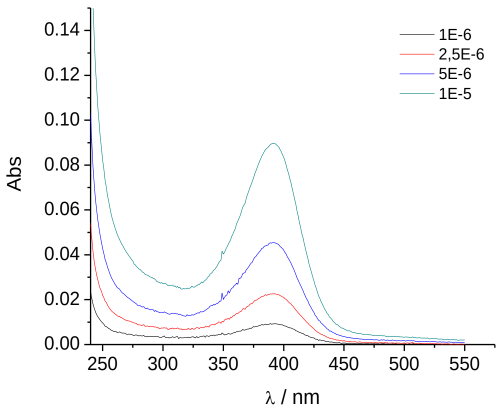

e)

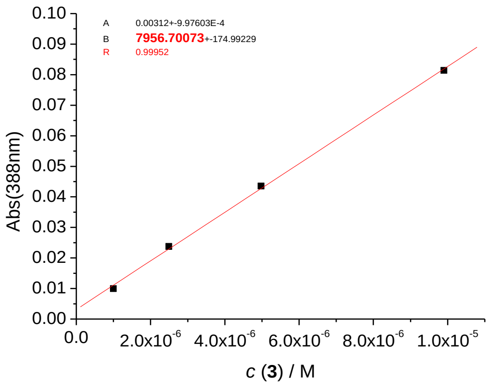

f)

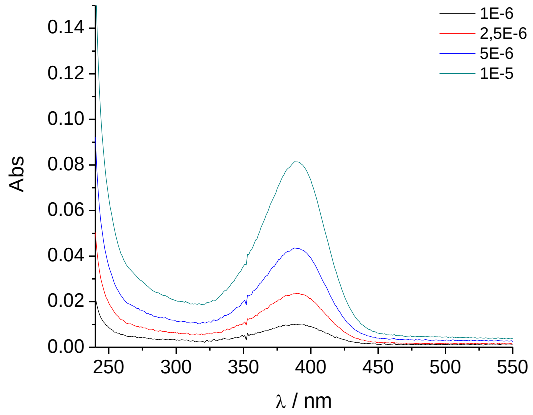

g)

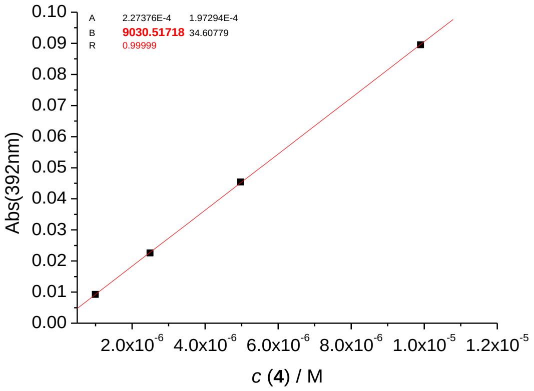

h)

Figure S1. a) dependence of UV/Vis spectra on concentration increase (1 ×10^{-6} M to 1 ×10^{-5} M) for **1**, b) calibration at λ_{max} = 403 nm (black) and λ_{max} = 276 nm for **1**, c) dependence of UV/Vis spectra on concentration increase for **2**, d) calibration at λ_{max} = 402 nm (black) and λ_{max} = 286 nm (red) for **2**, e) dependence of UV/Vis spectra on concentration increase for **3**, f) calibration at λ_{max} = 388 nm for **3**, g) dependence of UV/Vis spectra on concentration increase for **4**, h) calibration at λ_{max} = 392 nm for **4**.

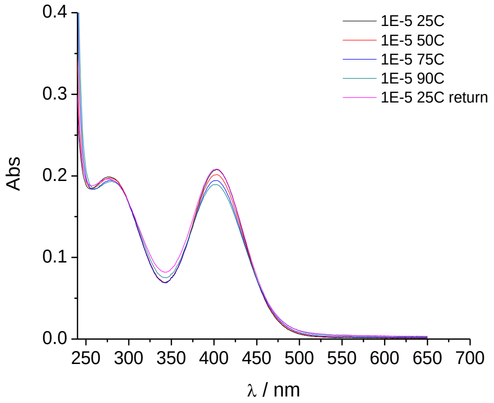

a)

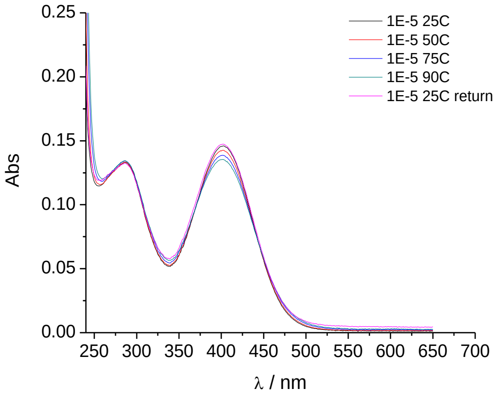

b)

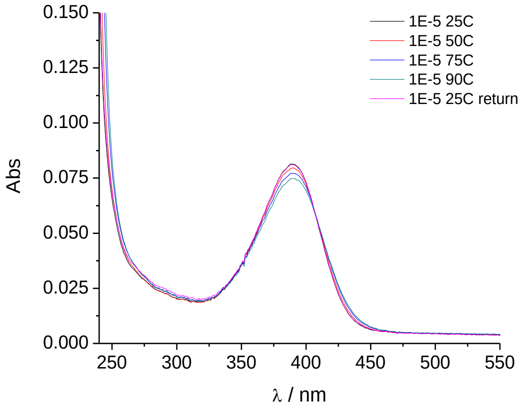

c)

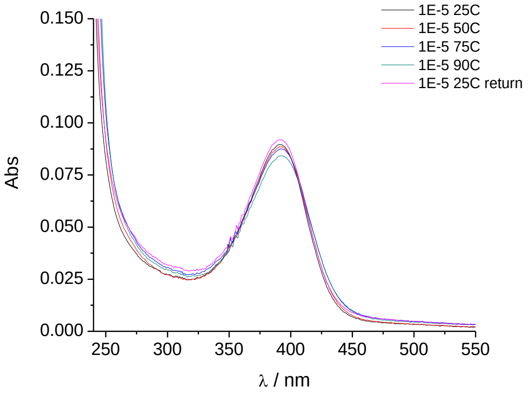

d)

Figure S2. a) dependence of UV/Vis spectra of **1** (c = 1 ×10^{-5} M) on temperature increase, b) dependence of UV/Vis spectra of **2** (c = 1 ×10^{-5} M) on temperature increase, c) dependence

of UV/Vis spectra of **3** (c = 1 × 10^{-5} M) on temperature increase, d) dependence of UV/Vis spectra of **4** (c = 1 × 10^{-5} M) on temperature increase.

2. Interactions with ds-DNA/RNA

2.1. UV/Vis titrations

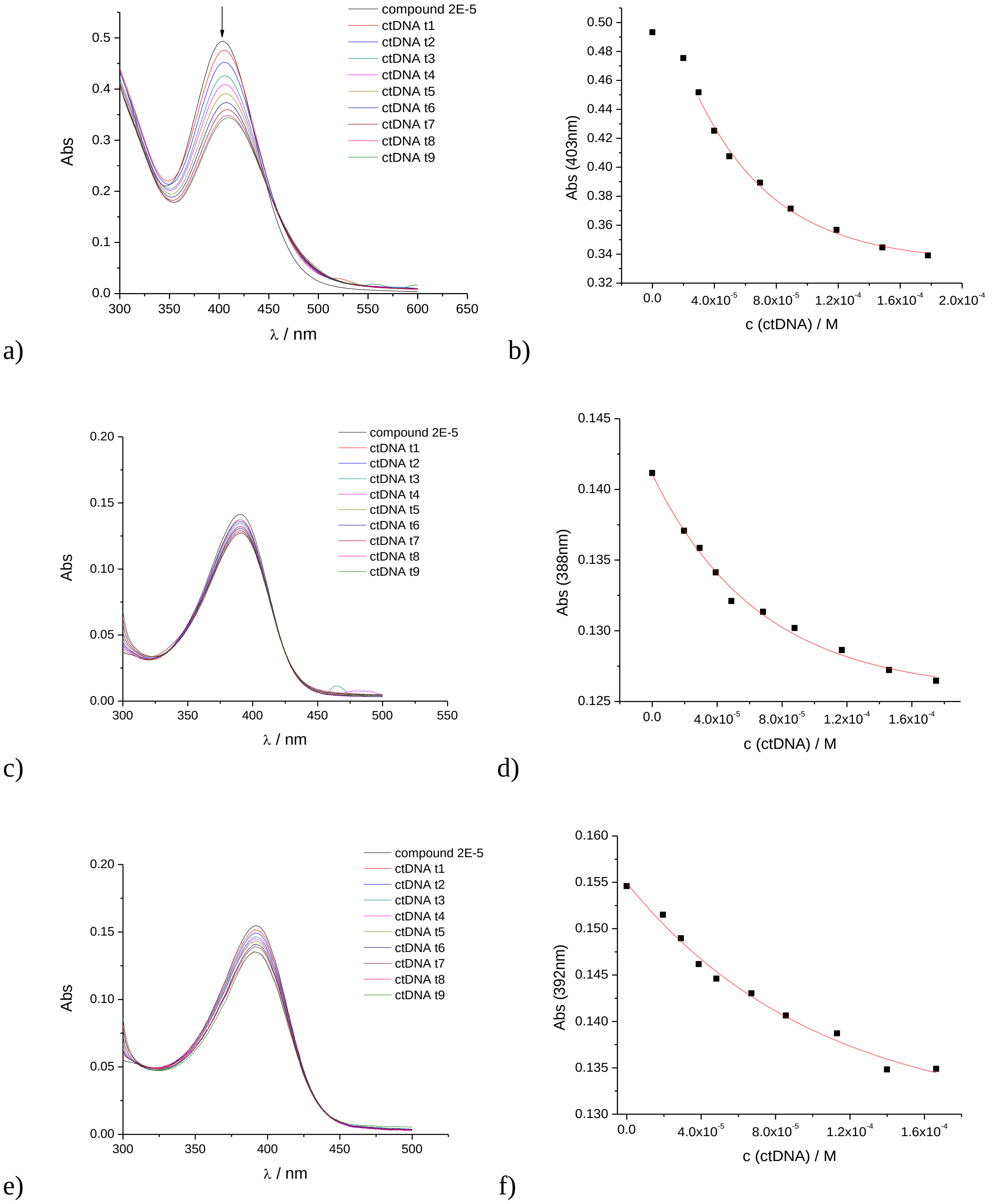

Figure S3. a) UV/Vis spectra of **1** ($c = 2 \times 10^5$ M) upon titration with ct-DNA, b) dependence of **1** absorbance at λ_{max} = 403 nm on *c*(pApU), c) UV/Vis spectra of **3** ($c = 2 \times 10^{-5}$ M) upon titration with ct-DNA, d) dependence of **3** absorbance at λ_{max} = 388 nm on *c*(pApU), c)
UV/Vis spectra of **4** ($c = 2 \times 10^5$ M) upon titration with ct-DNA, f) dependence of **4** absorbance at λ_{max} = 392 nm on *c*(pApU).

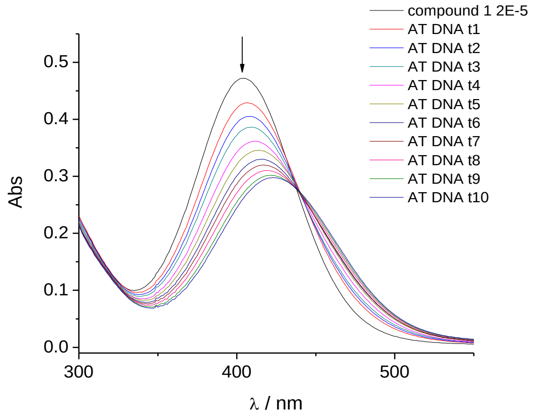

a)

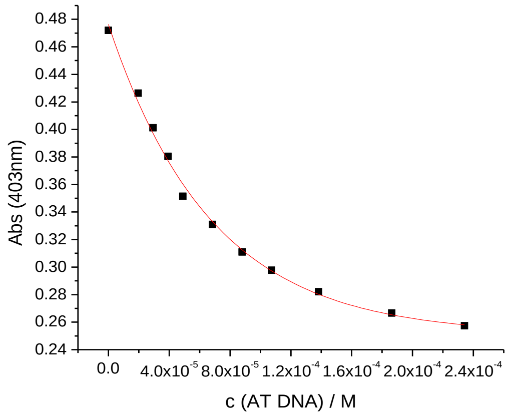

b)

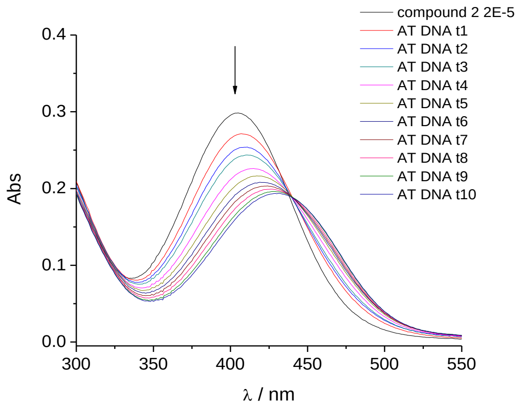

c)

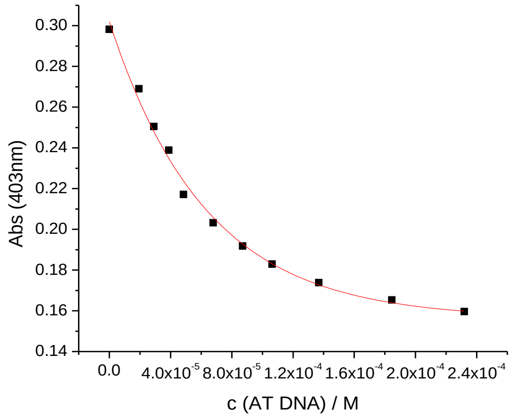

d)

Figure S4.) UV/Vis spectra of 1 (c = 2 × 10-5 M) upon titration with AT DNA, b) dependence of 1 absorbance at λmax = 403 nm on c(AT DNA), c) UV/Vis spectra of 2 (c = 2 × 10-5 M) upon titration with AT DNA, d) dependence of 2 absorbance at λmax = 403 nm on c(AT DNA).

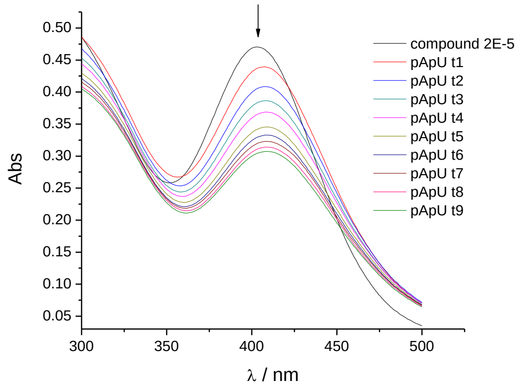

a)

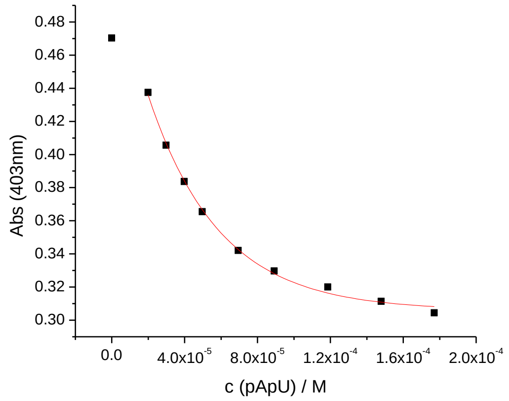

b)

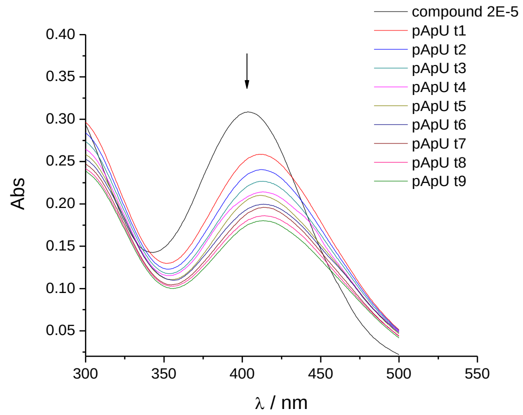

c)

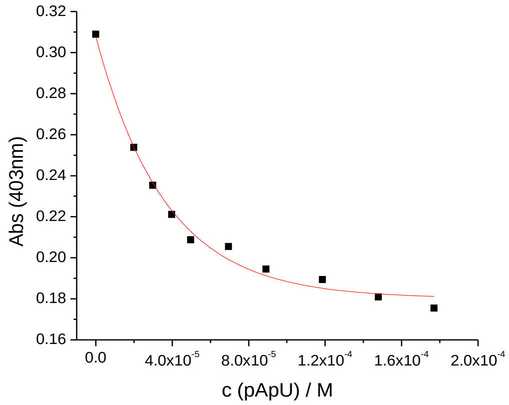

d)

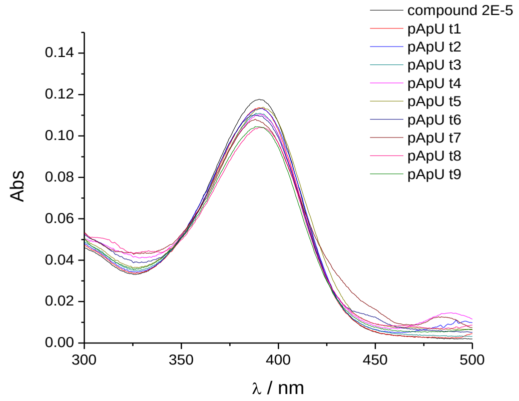

e)

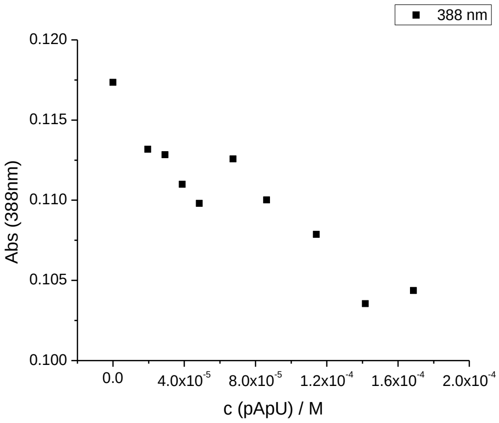

f)

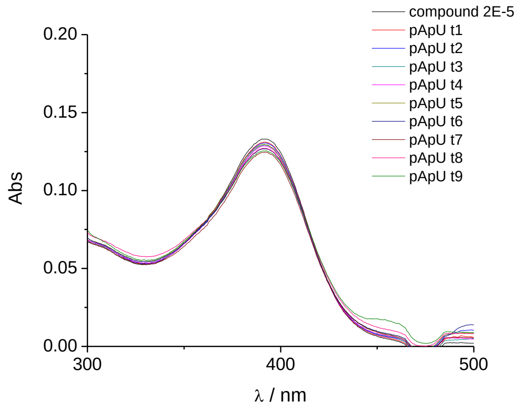

g)

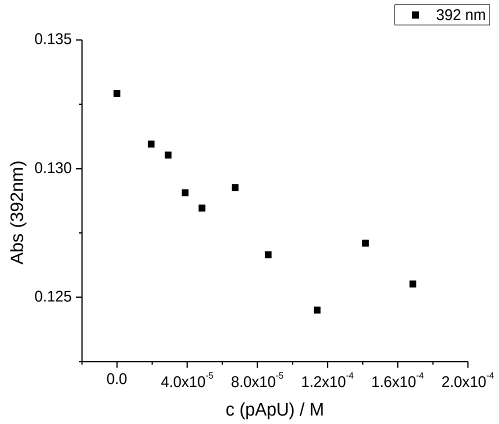

h)

Figure S5. a) UV/Vis spectra of **1** ($c = 2 \times 10^{-5}$ M) upon titration with pApU, b) dependence of **1** absorbance at λ_{max} = 403 nm on c(pApU), c) UV/Vis spectra of **2** ($c = 2 \times 10^{-5}$ M) upon titration with pApU, d) dependence of **2** absorbance at λ_{max} = 403 nm on c(pApU), e) UV/Vis spectra of **3** ($c = 2 \times 10^{-5}$ M) upon titration with pApU, f) dependence of **3** absorbance at λ_{max} = 388 nm on c(pApU), h) UV/Vis spectra of **4** ($c = 2 \times 10^{-5}$ M) upon titration with pApU, g) dependence of **4** absorbance at λ_{max} = 392 nm on c(pApU), Done at pH = 7, sodium cacodylate buffer, I = 0.05 M.

2.2. Circular dichroism experiments

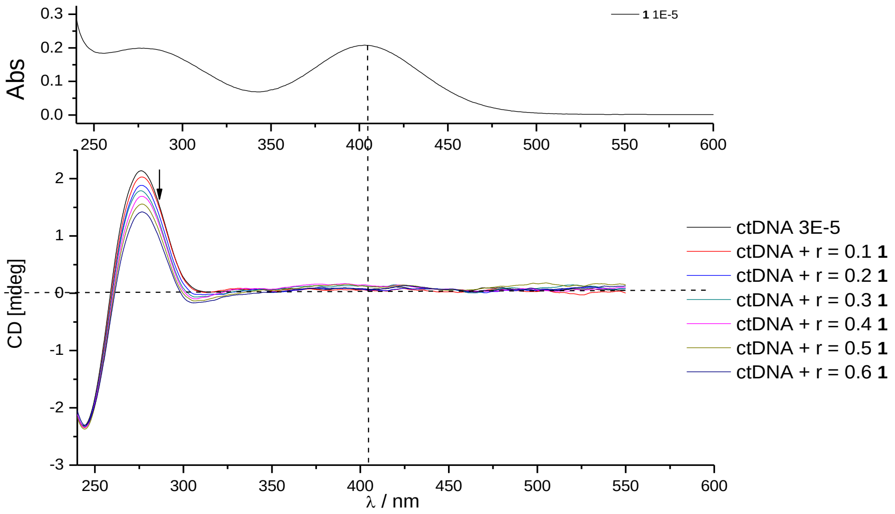

Figure S6. CD titration of ctDNA (c = 3 ×10^{-5} M) with **1** at molar ratios r = [compound] / [ctDNA] (pH 7.0, buffer sodium cacodylate, I = 0.05 M).

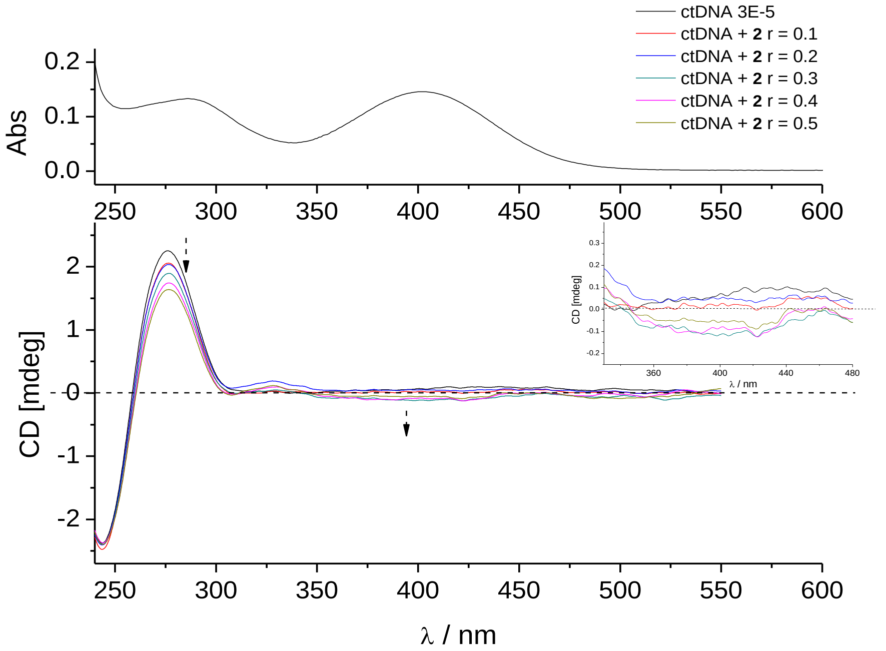

Figure S7. CD titration of ctDNA (c = 3 × 10^{5} M) with **2** at molar ratios r = [compound] / [ctDNA] (pH 7.0, buffer sodium cacodylate, I = 0.05 M), inset: ICD at λ = 410 nm.

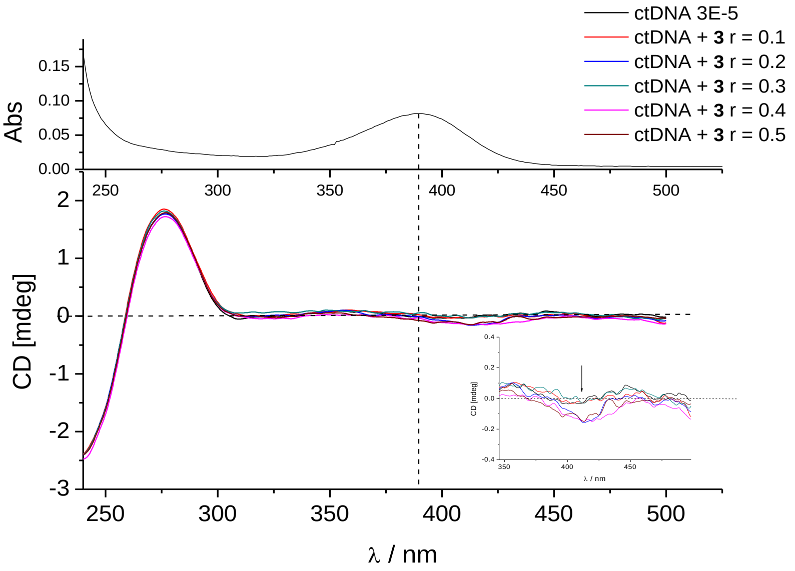

Figure S8. CD titration of ctDNA (c = 3 × 10^{5} M) with **3** at molar ratios r = [compound] / [ctDNA] (pH 7.0, buffer sodium cacodylate, I = 0.05 M), inset: ICD at λ = 414 nm.

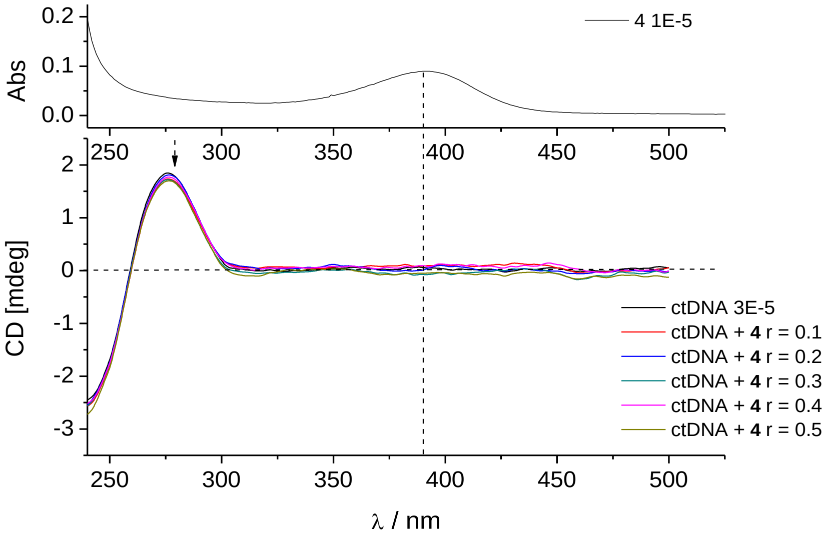

Figure S9. CD titration of ctDNA (c = 3 ×10^{-5} M) with **4** at molar ratios r = [compound] / [ctDNA] (pH 7.0, buffer sodium cacodylate, I = 0.05 M).

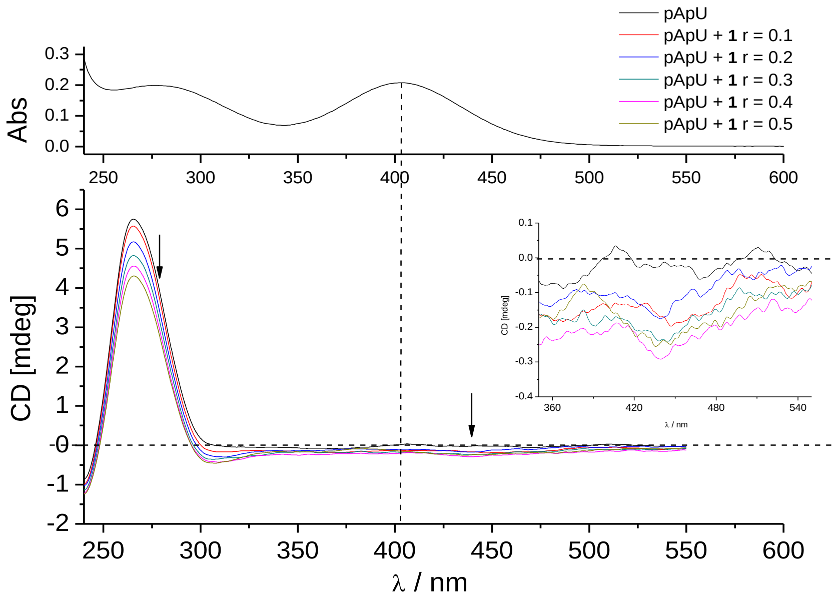

Figure S10. CD titration of pApU (c = 3 × 10^{-5} M) with **1** at molar ratios r = [compound] / [pApU] (pH 7.0, buffer sodium cacodylate, I = 0.05 M), inset: ICD at λ = 440 nm.

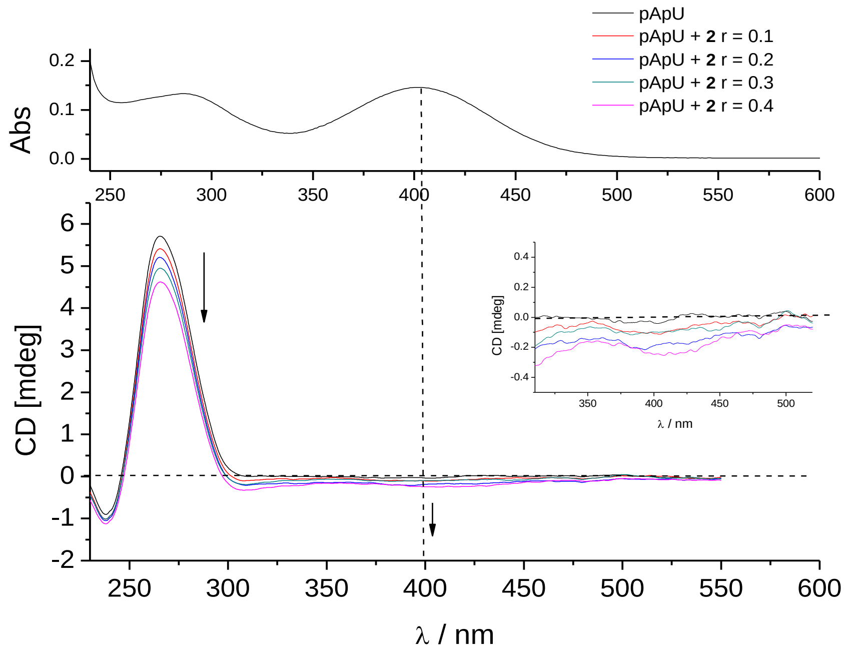

Figure S11. CD titration of pApU (c = 3 × 10^{-5} M) with **2** at molar ratios r = [compound] / [pApU] (pH 7.0, buffer sodium cacodylate, I = 0.05 M), inset: ICD at λ = 410 nm.

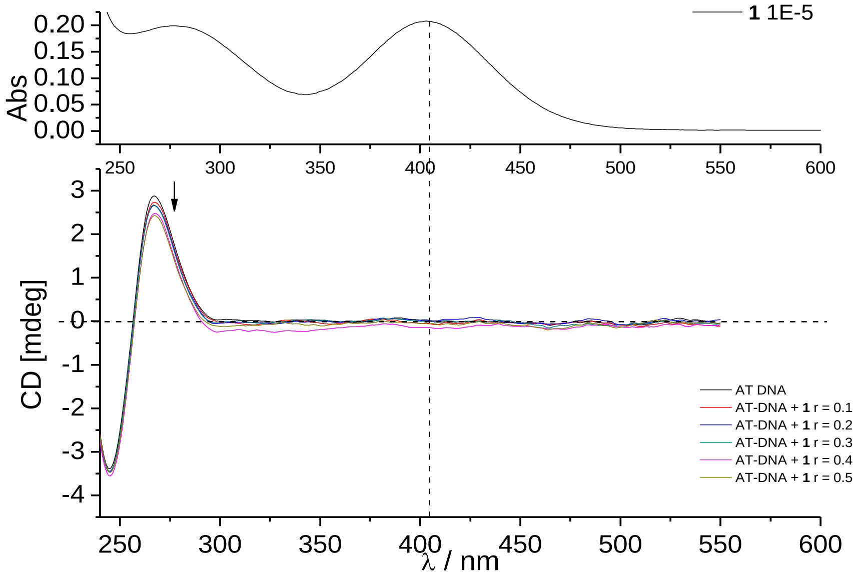

Figure S12. CD titration of AT-DNA ($c = 3 \times 10^{-5}$ M) with **1** at molar ratios r = [compound] / [AT-DNA] (pH 7.0, buffer sodium cacodylate, I = 0.05 M).

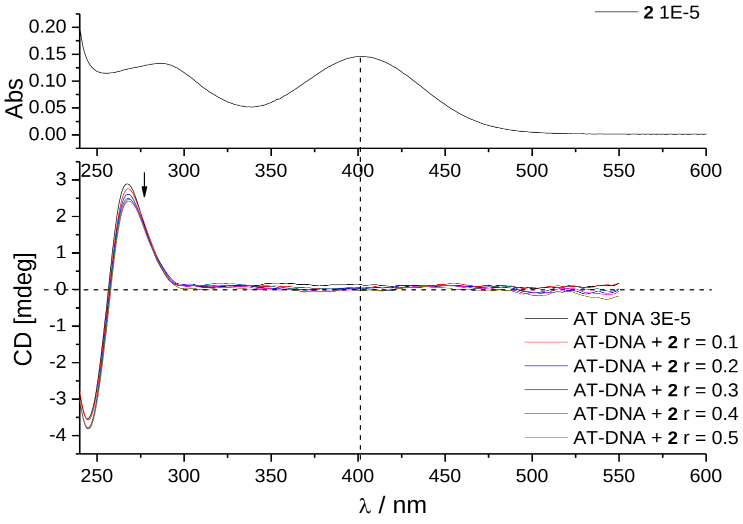

Figure S13. CD titration of AT-DNA ($c = 3 \times 10^{-5}$ M) with **2** at molar ratios r = [compound] / [AT-DNA] (pH 7.0, buffer sodium cacodylate, I = 0.05 M).

2.3. Thermal denaturation measurements

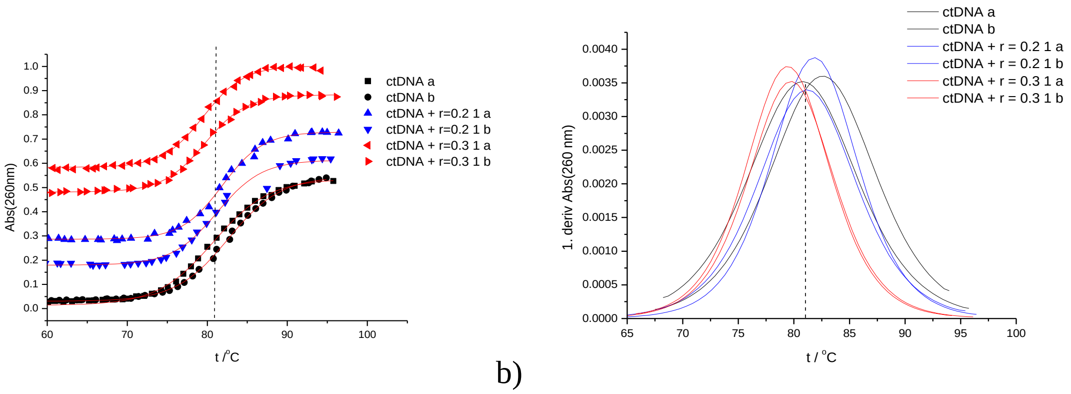

a) b)

Figure S14. a) Melting curves of ct-DNA upon addition r = 0.2 or r = 0.3 ([compound]/[polynucleotide]) of **1** at pH 7.0, (buffer sodium cacodylate, I = 0.05 M), red lines denote fitting of experimental data to sigmoidal eq. by Origin 7.5., b) the first derivation of absorbance (fitted to sigmoidal eq.) on temperature. Absorbance values are normalized by maximum absorbance and offset for better visualization of data.

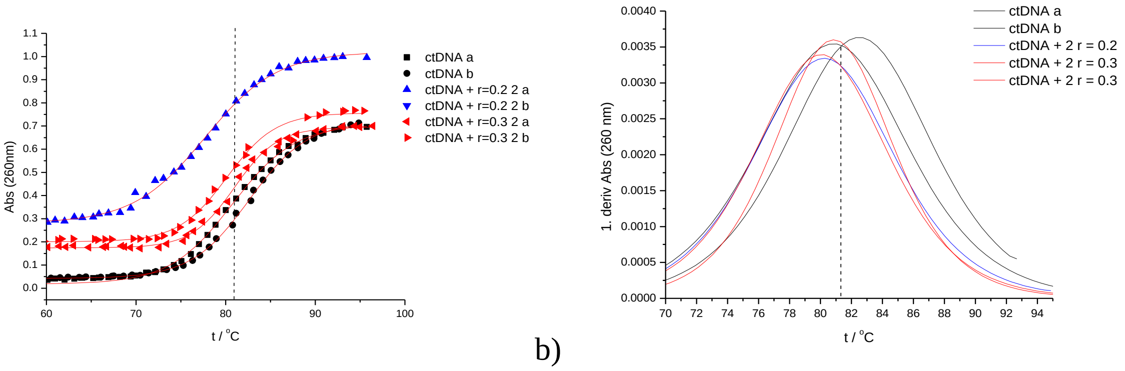

a) b)

Figure S15. a) Melting curves of ctDNA upon addition r = 0.3 or r = 0.2 ([compound]/[polynucleotide]) of **2** at pH 7.0, (buffer sodium cacodylate, I = 0.05 M), red lines denote fitting of experimental data to sigmoidal eq. by Origin 7.5., b) the first derivation of absorbance (fitted to sigmoidal eq.) on temperature. Absorbance values are normalized by maximum absorbance and offset for better visualization of data.

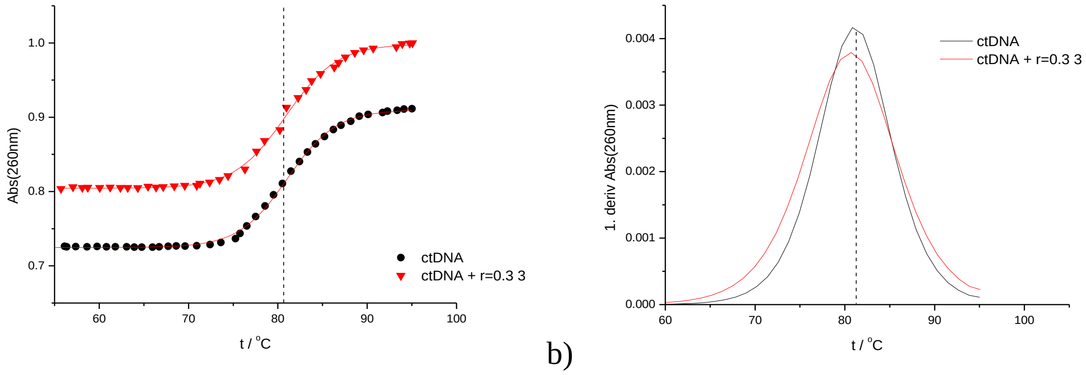

a) b)

Figure S16. a) Melting curves of ct-DNA upon addition r = 0.3 ([compound]/[polynucleotide]) of **3** at pH 7.0, (buffer sodium cacodylate, I = 0.05 M), red lines denote fitting of experimental

data to sigmoidal eq. by Origin 7.5, b) the first derivation of absorbance (fitted to sigmoidal eq.) on temperature. Absorbance values are normalized by maximum absorbance and offset for better visualization of data.

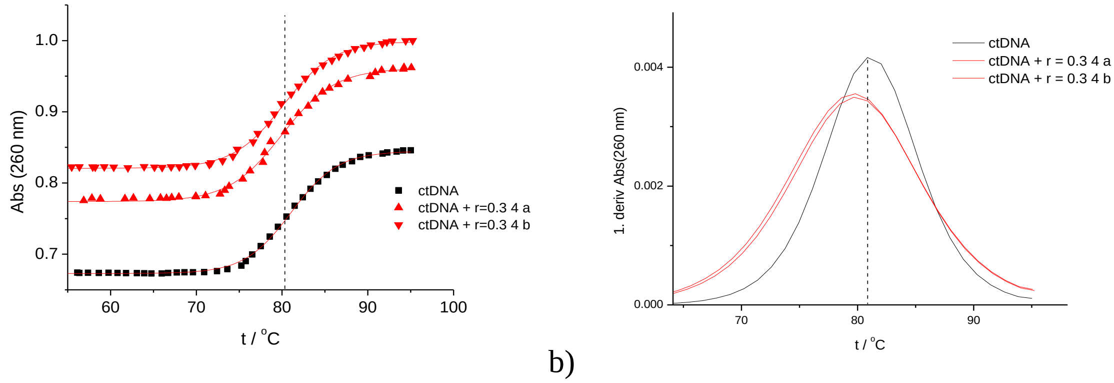

a) b)

Figure S17. a) Melting curves of ct-DNA upon addition r = 0.3 ([compound]/[polynucleotide]) of **4** at pH 7.0, (buffer sodium cacodylate, I = 0.05 M), red lines denote fitting of experimental data to sigmoidal eq. by Origin 7.5, b) the first derivation of absorbance (fitted to sigmoidal eq.) on temperature. Absorbance values are normalized by maximum absorbance and offset for better visualization of data.

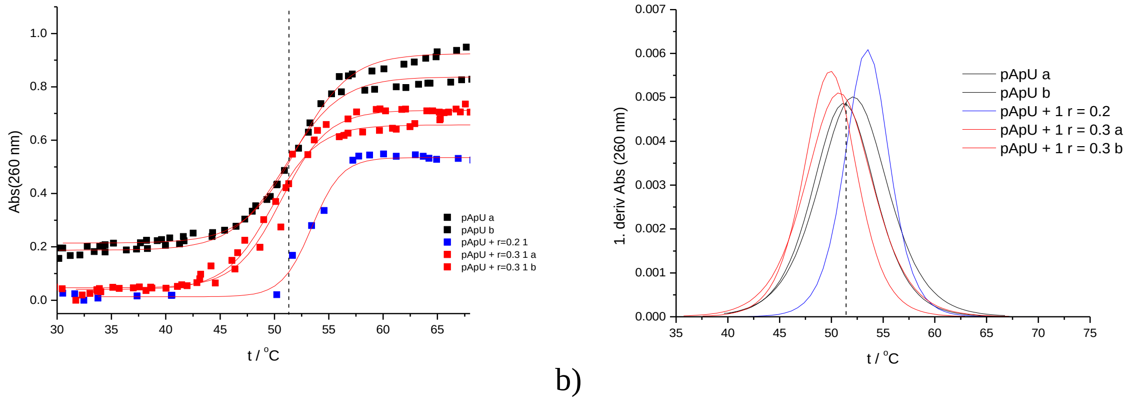

a) b)

Figure S18. a) Melting curves of pApU upon addition r = 0.2 or r = 0.3 ([compound]/[polynucleotide]) of **1** at pH 7.0, (buffer sodium cacodylate, I = 0.05 M), red lines denote fitting of experimental data to sigmoidal eq. by Origin 7.5., b) the first derivation of absorbance (fitted to sigmoidal eq.) on temperature. Absorbance values are normalized by maximum absorbance and offset for better visualization of data.

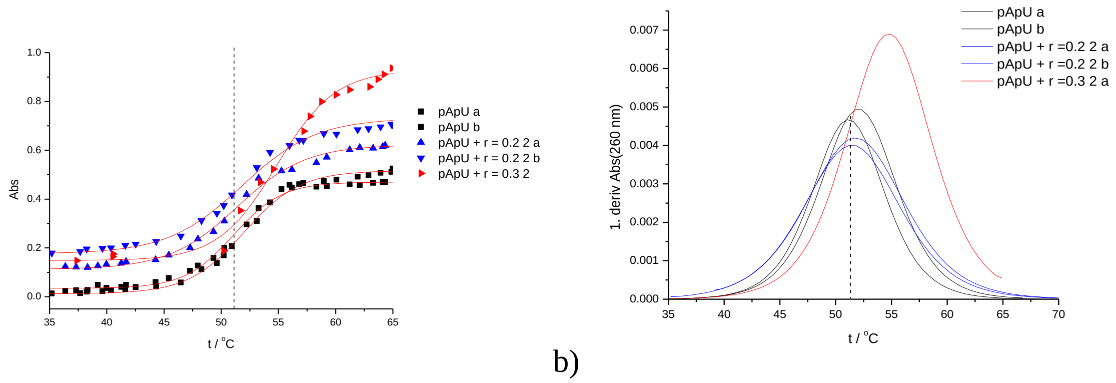

a) b)

Figure S19. a) Melting curves of pApU upon addition r = 0.2 or r = 0.3 p([compound]/[polynucleotide]) of **2** at pH 7.0, (buffer sodium cacodylate, *I* = 0.05 M), red lines denote fitting of experimental data to sigmoidal eq. by Origin 7.5., b) the first derivation of absorbance (fitted to sigmoidal eq.) on temperature. Absorbance values are normalized by maximum absorbance and offset for better visualization of data.

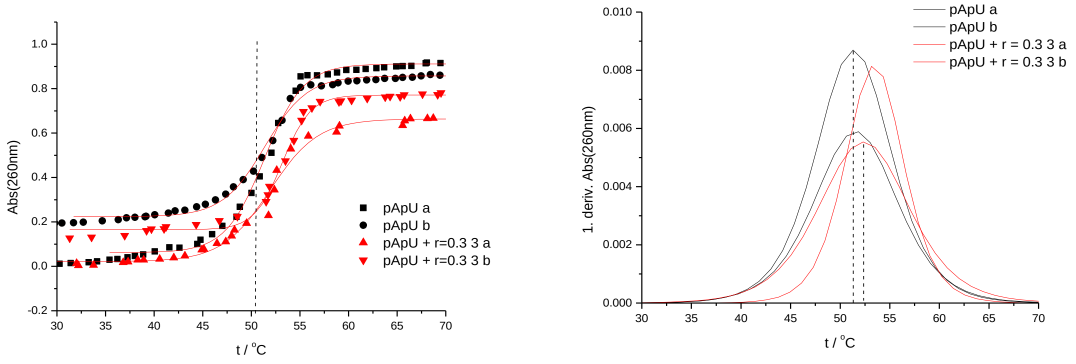

a) b)

Figure S20. a) Melting curves of pApU upon addition r = 0.3 ([compound]/[polynucleotide]) of **3** at pH 7.0, (buffer sodium cacodylate, *I* = 0.05 M), red lines denote fitting of experimental data to sigmoidal eq. by Origin 7.5, b) the first derivation of absorbance (fitted to sigmoidal eq.) on temperature. Absorbance values are normalized by maximum absorbance and offset for better visualization of data.

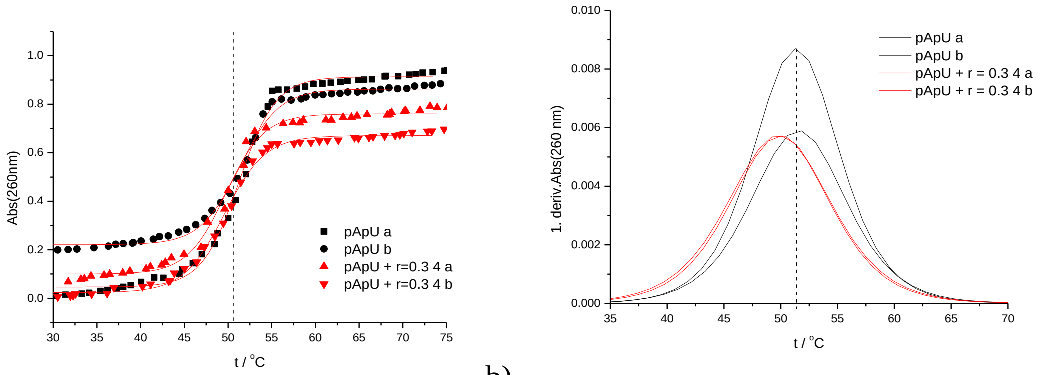

a) b)

Figure S21. Melting curves of pApU upon addition r = 0.3 ([compound]/[polynucleotide]) of **4** at pH 7.0, (buffer sodium cacodylate, *I* = 0.05 M), red lines denote fitting of experimental data to sigmoidal eq. by Origin 7.5, b) the first derivation of absorbance (fitted to sigmoidal eq.) on temperature. Absorbance values are normalized by maximum absorbance and offset for better visualization of data.

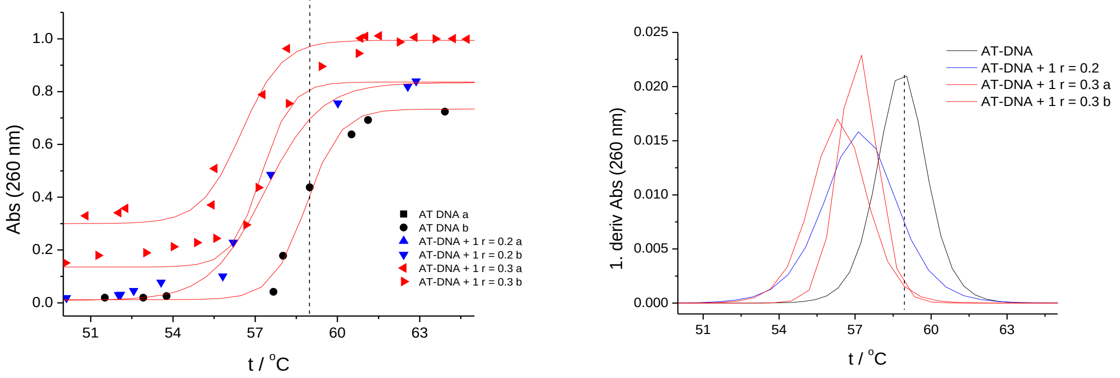

a) b)

Figure S22. a) Melting curves of AT-DNA upon addition r = 0.2 or r = 0.3 p([compound]/[polynucleotide]) of **1** at pH 7.0, (buffer sodium cacodylate, *I* = 0.05 M), red lines denote fitting of experimental data to sigmoidal eq. by Origin 7.5., b) the first derivation of absorbance (fitted to sigmoidal eq.) on temperature. Absorbance values are normalized by maximum absorbance and offset for better visualization of data.

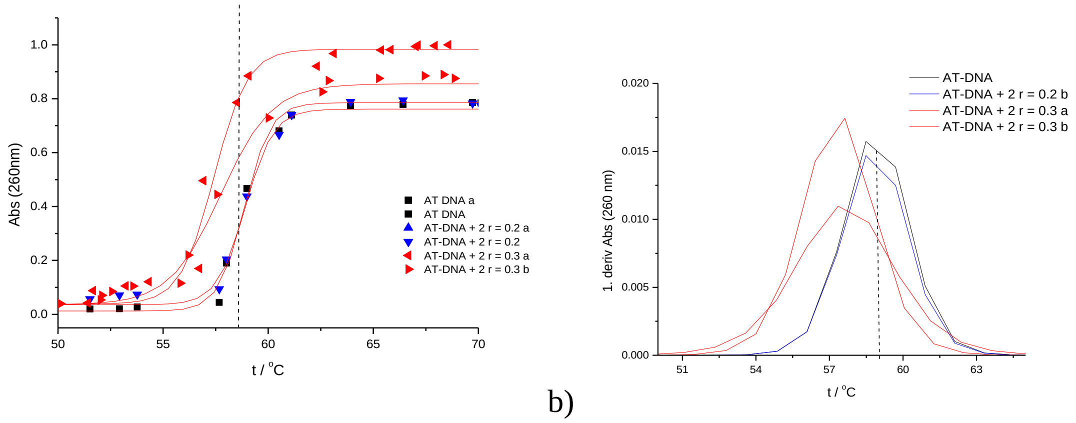

a) b)

Figure S23. a) Melting curves of AT-DNA upon addition r = 0.2 or r = 0.3 p([compound]/[polynucleotide]) of **2** at pH 7.0, (buffer sodium cacodylate, *I* = 0.05 M), red lines denote fitting of experimental data to sigmoidal eq. by Origin 7.5., b) the first derivation of absorbance (fitted to sigmoidal eq.) on temperature. Absorbance values are normalized by maximum absorbance and offset for better visualization of data.

3. NMR spectra of benzothiazines

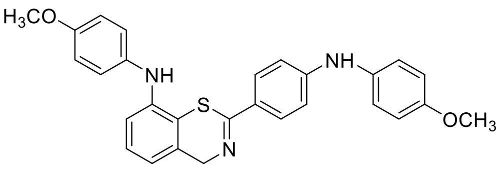

1'

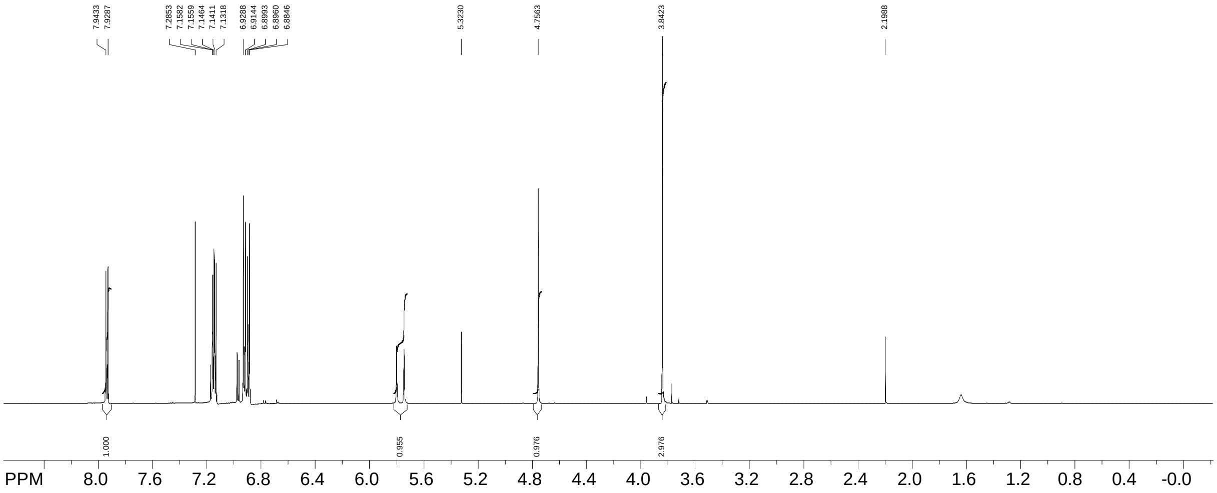

file: C:\Users\Irena\Desktop\000 PUBLIKACIJA\NMR spektri benztiazini\cpd4-raw nmr\10\fid expt: <zg30>
transmitter freq.: 600.135401 MHz
time domain size: 131072 points
width: 12019.23 Hz = 20.027532 ppm = 0.091699 Hz/pt
number of scans: 64

freq. of 0 ppm: 600.130000 MHz
processed size: 65536 complex points
LB: 0.000 GB: 0.0000

Figure S24. ^{1}H NMR ($CDCl_3$) spectrum of benzothiazine **1'**.

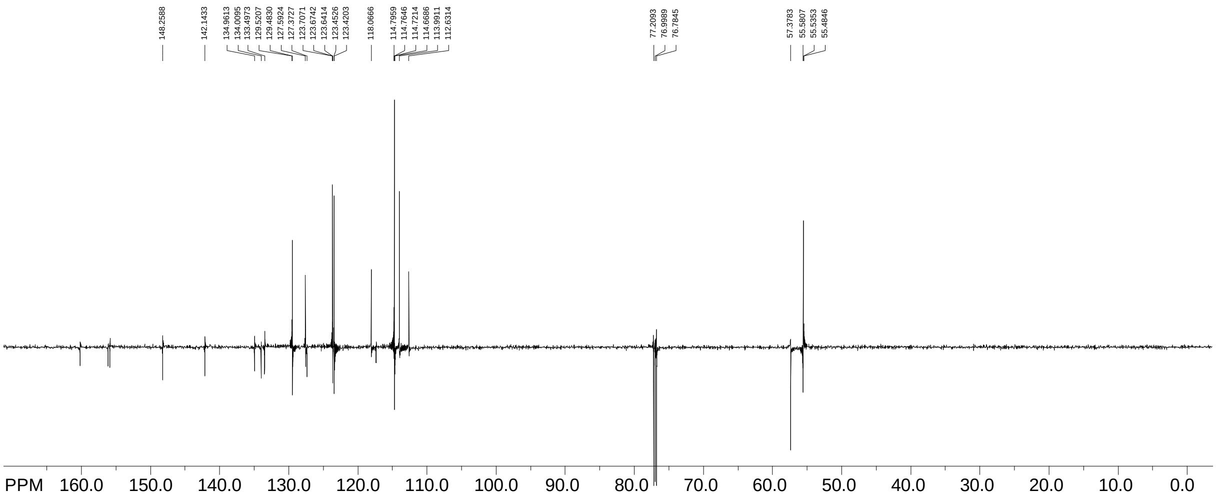

file: C:\Users\Irena\Desktop\000 PUBLIKACIJA\NMR spektri benztiazini\cpd4-raw nmr\11\fid expt: <deptqgpsp.2>
transmitter freq.: 150.921671 MHz
time domain size: 16384 points
width: 39062.50 Hz = 258.826315 ppm = 2.384186 Hz/pt
number of scans: 12500

freq. of 0 ppm: 150.902815 MHz
processed size: 16384 complex points
LB: 0.000 GB: 0.0000

Figure S25. ^{13}C NMR ($CDCl_3$) spectrum of benzothiazine **1'**.

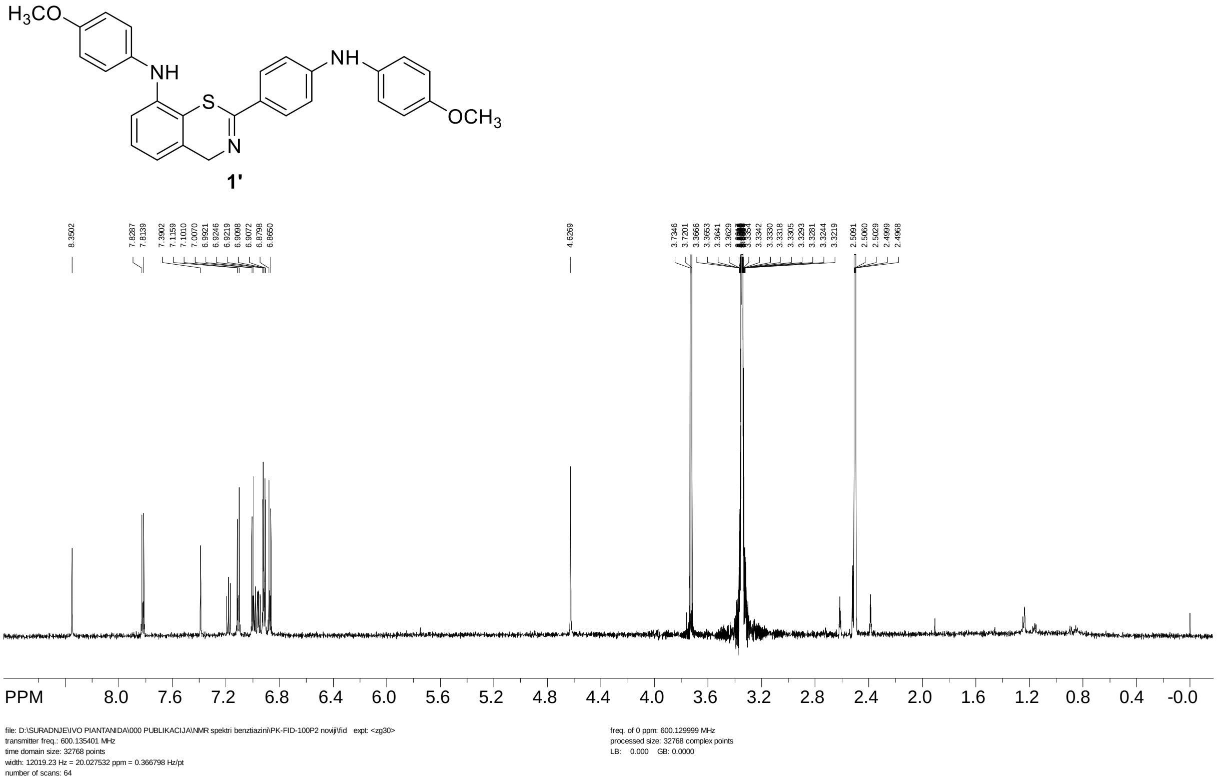

Figure S26. ^{1}H NMR (DMSO) spectrum of benzothiazine **1'**.

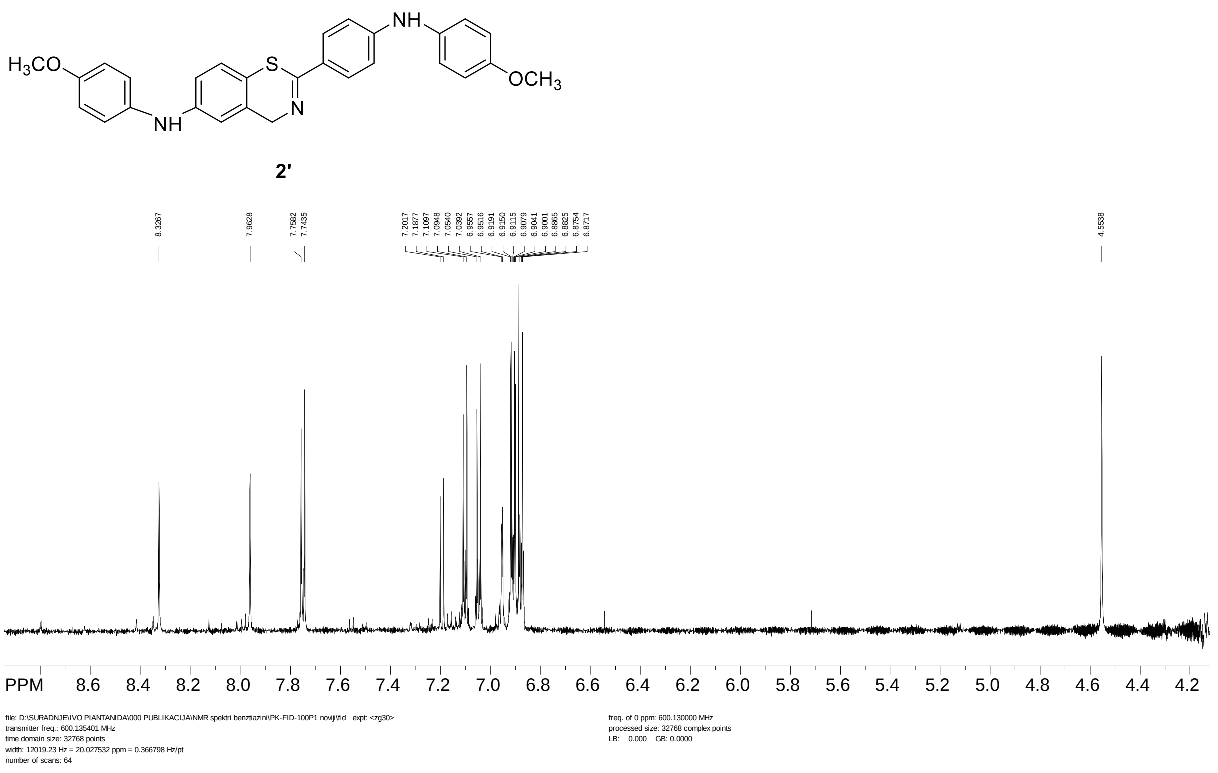

Figure S27. ^{1}H NMR (DMSO) spectrum of benzothiazine **2'**.

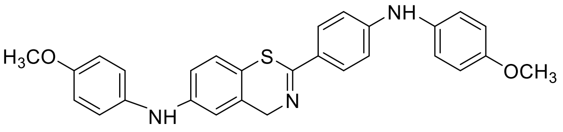

2'

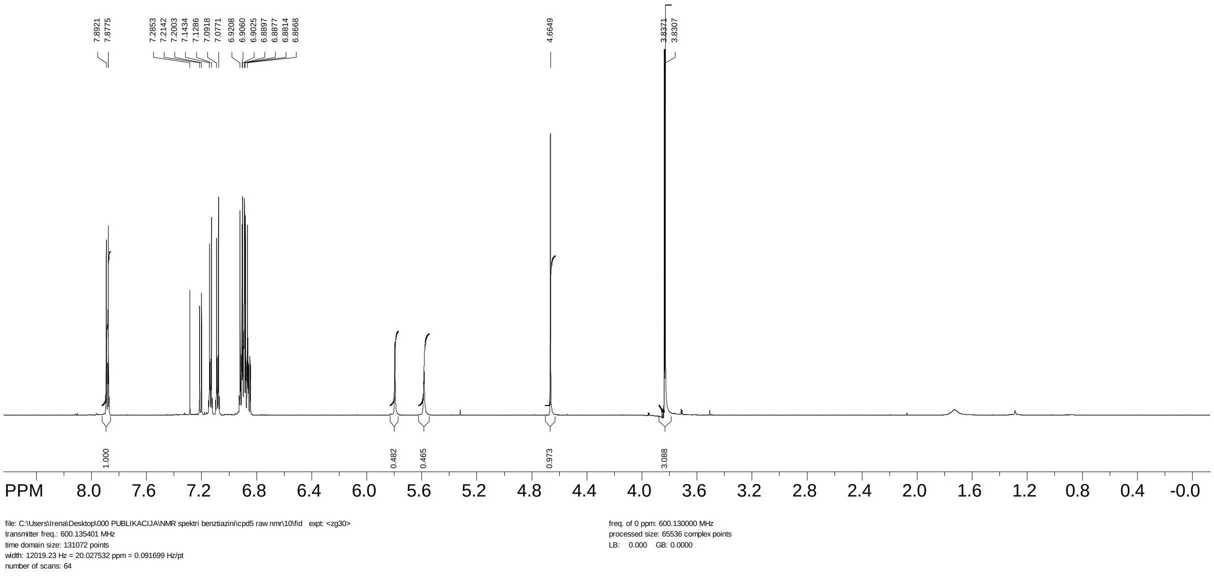

Figure S28. ^{1}H NMR ($CDCl_3$) spectrum of benzothiazine **2'**.

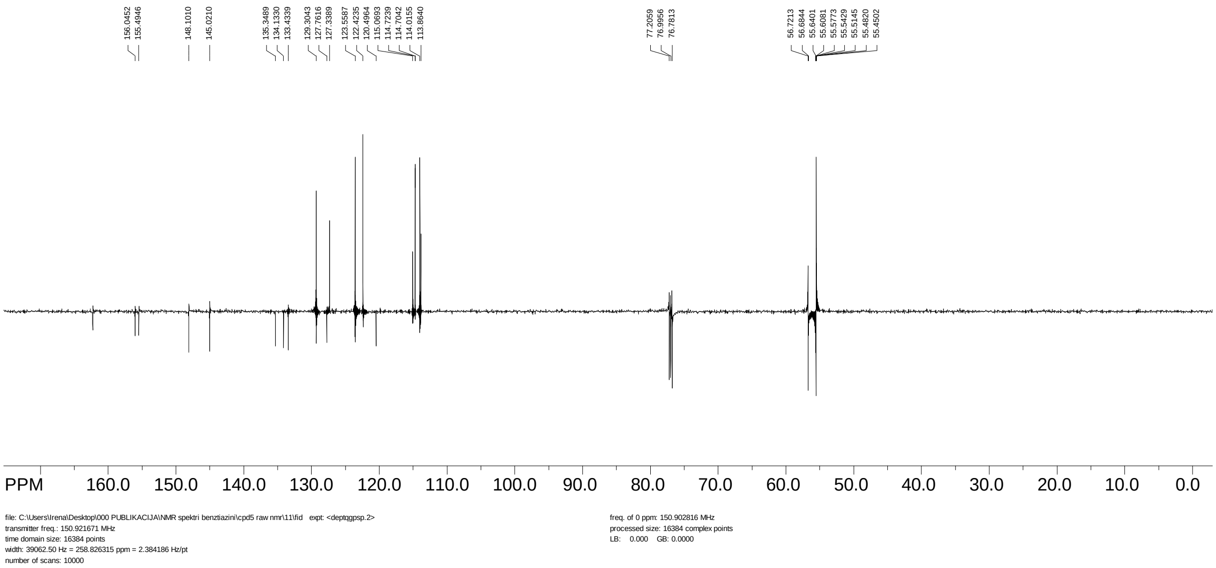

Figure S29. ^{13}C NMR ($CDCl_3$) spectrum of benzothiazine **2'**.

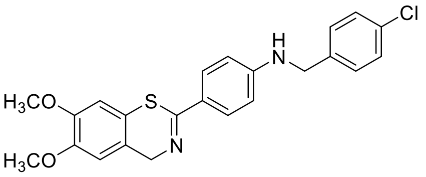

3'

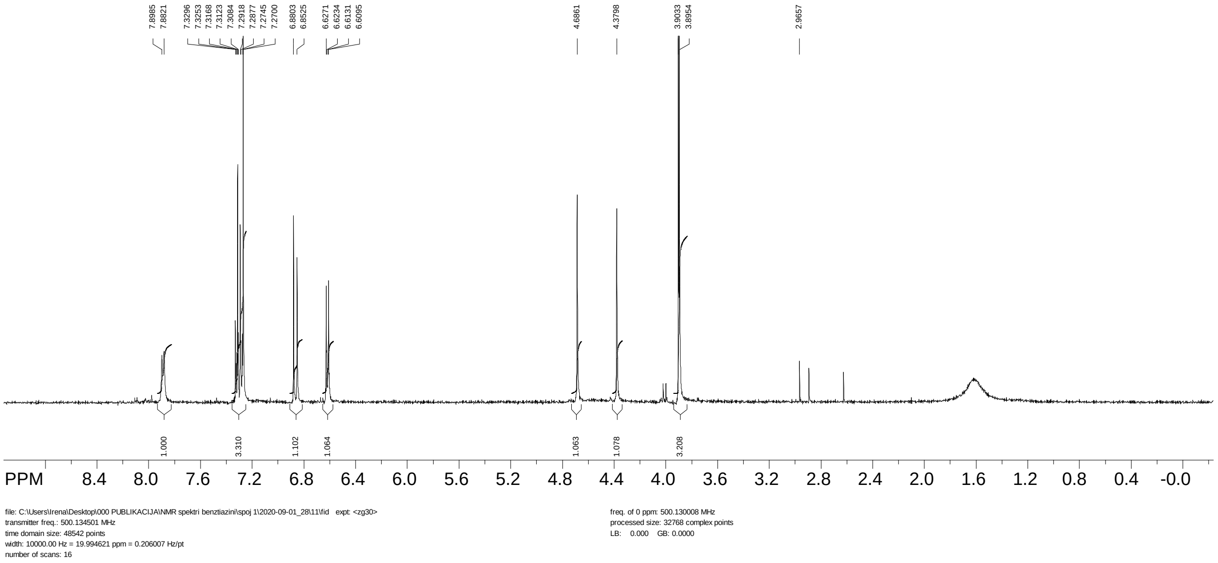

Figure S30. ^{1}H NMR ($CDCl_3$) spectrum of benzothiazine **3'**.

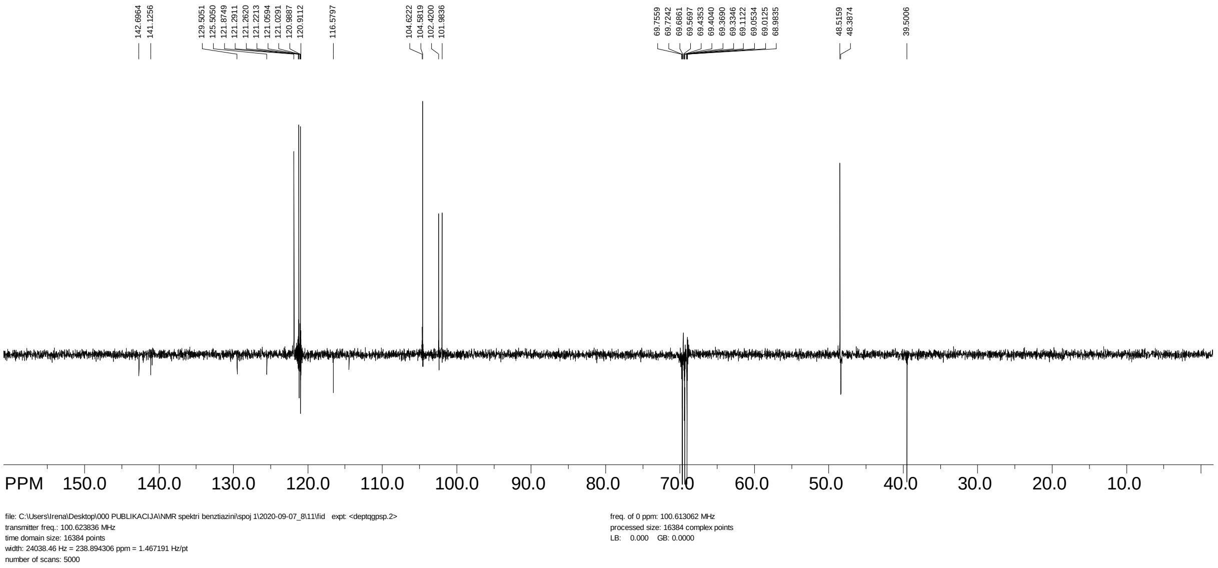

Figure S31. ^{13}C NMR ($CDCl_3$) spectrum of benzothiazine **3'**.

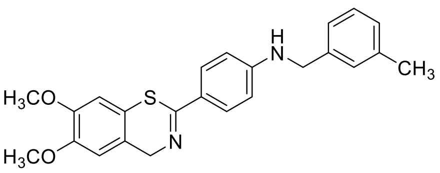

4'

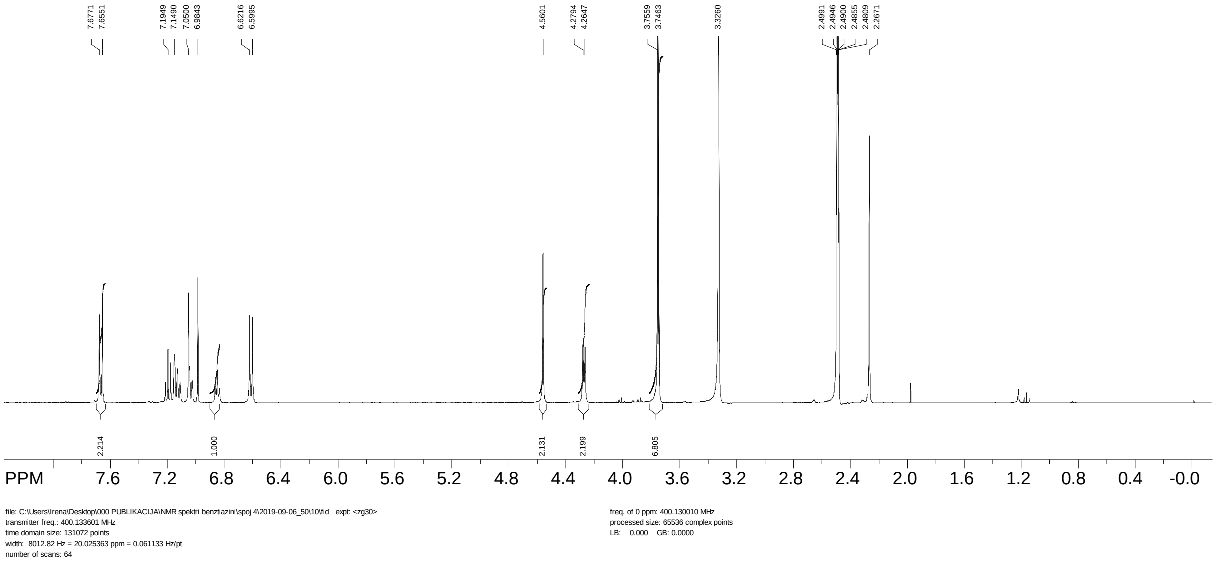

Figure S32. ^{1}H NMR (DMSO) spectrum of benzothiazine **4'**.

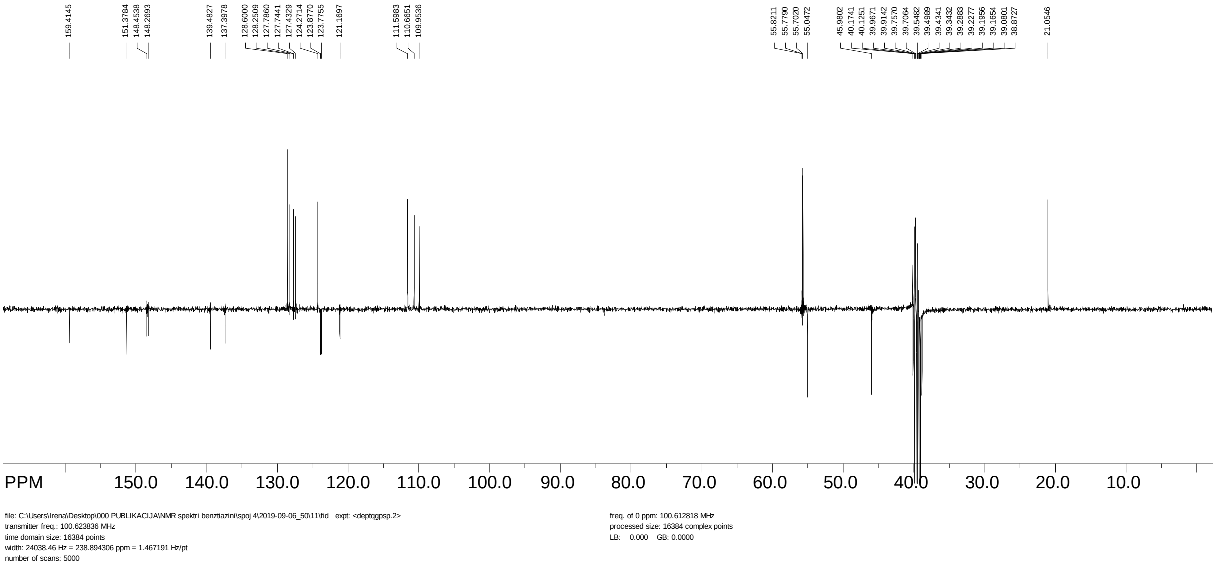

Figure S33. ^{13}C NMR (DMSO) spectrum of benzothiazine **4'**.

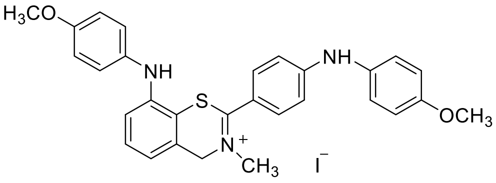

1

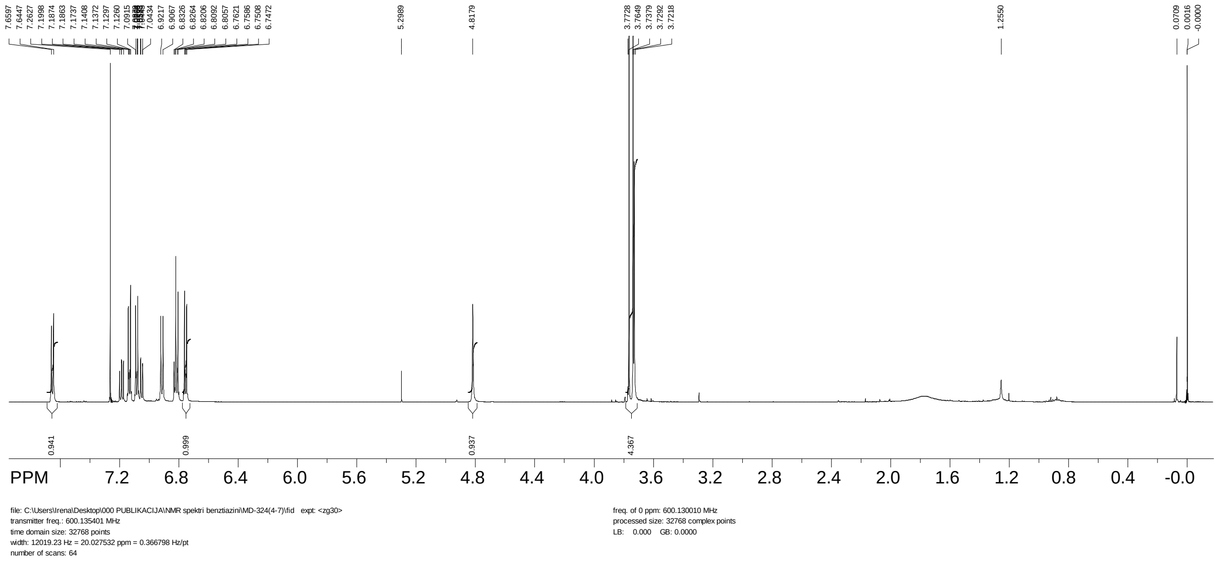

Figure S34. ^{1}H NMR ($CDCl_3$) spectrum of benzothiazine **1**.

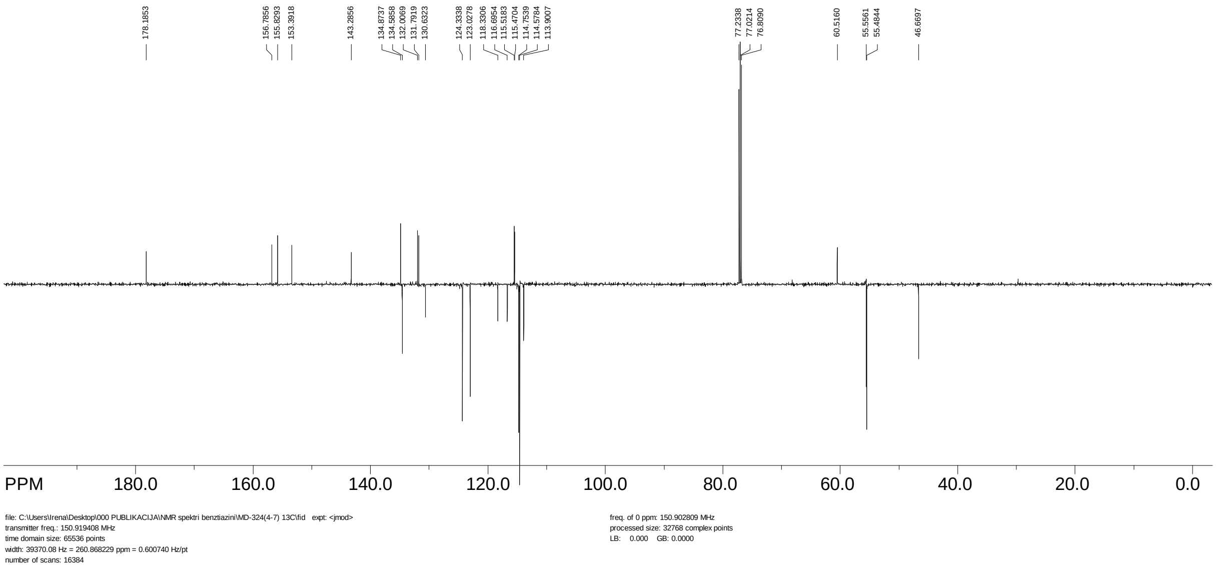

Figure S35. ^{13}C NMR ($CDCl_3$) spectrum of benzothiazine **1**.

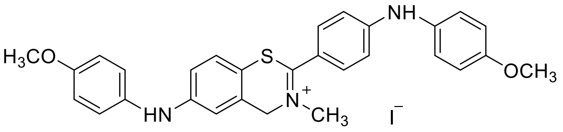

2

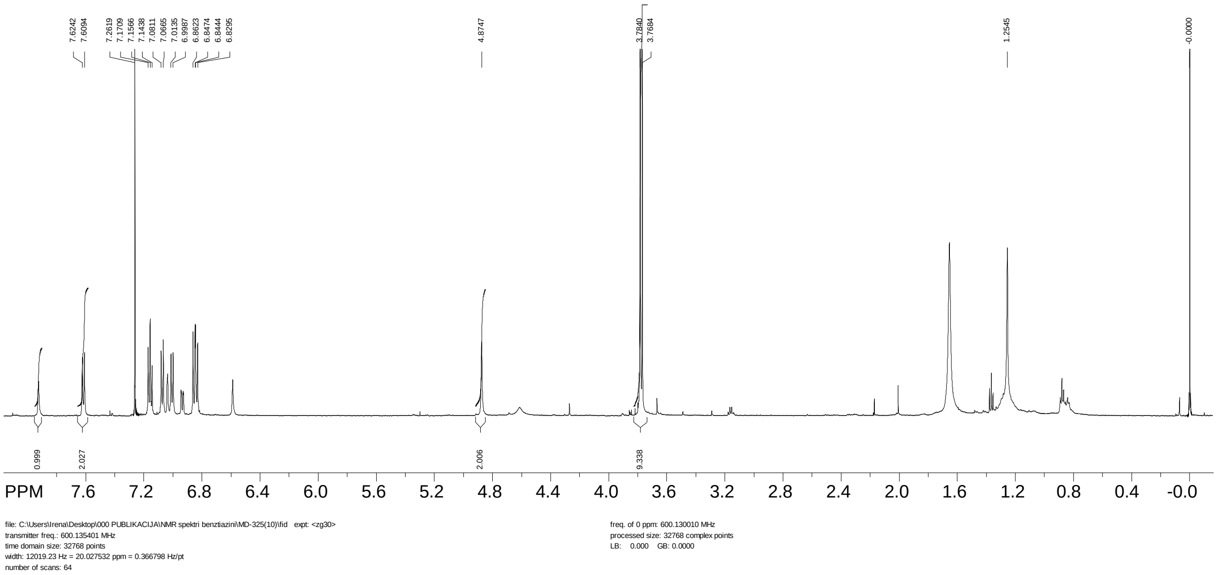

Figure S36. ^{1}H NMR ($CDCl_3$) spectrum of benzothiazine **2**.

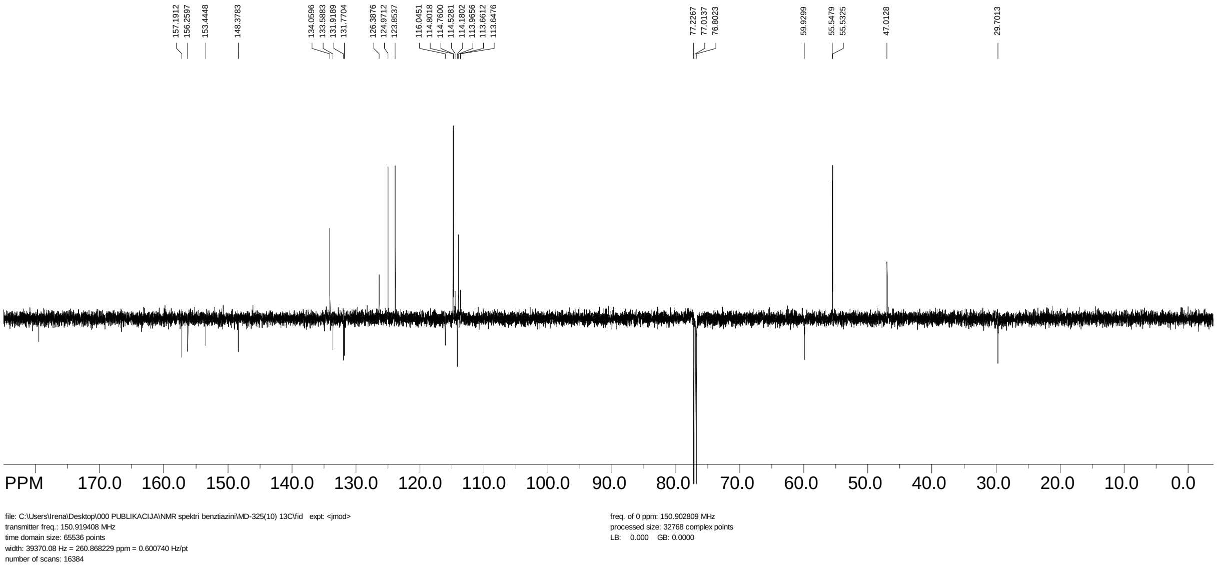

Figure S37. ^{13}C NMR ($CDCl_3$) spectrum of benzothiazine **2**.

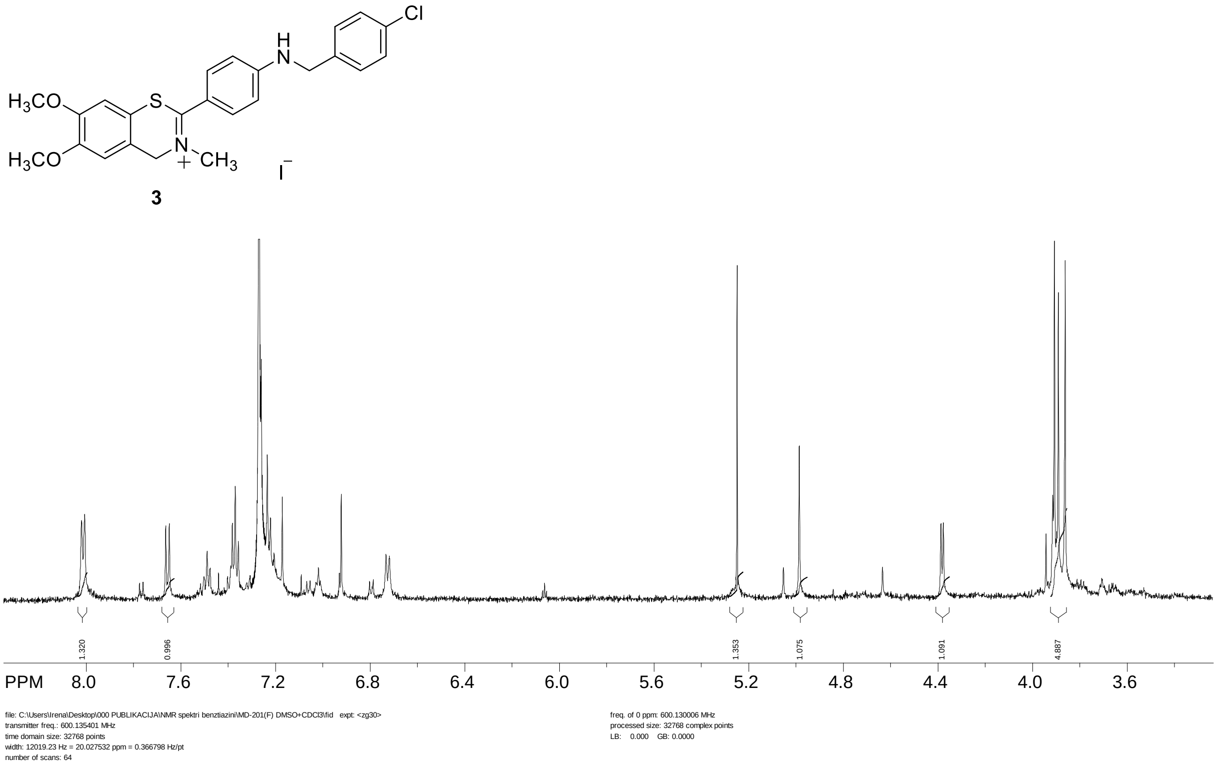

Figure S38. ^{1}H NMR ($CDCl_3$ + DMSO) spectrum of benzothiazine **3**.

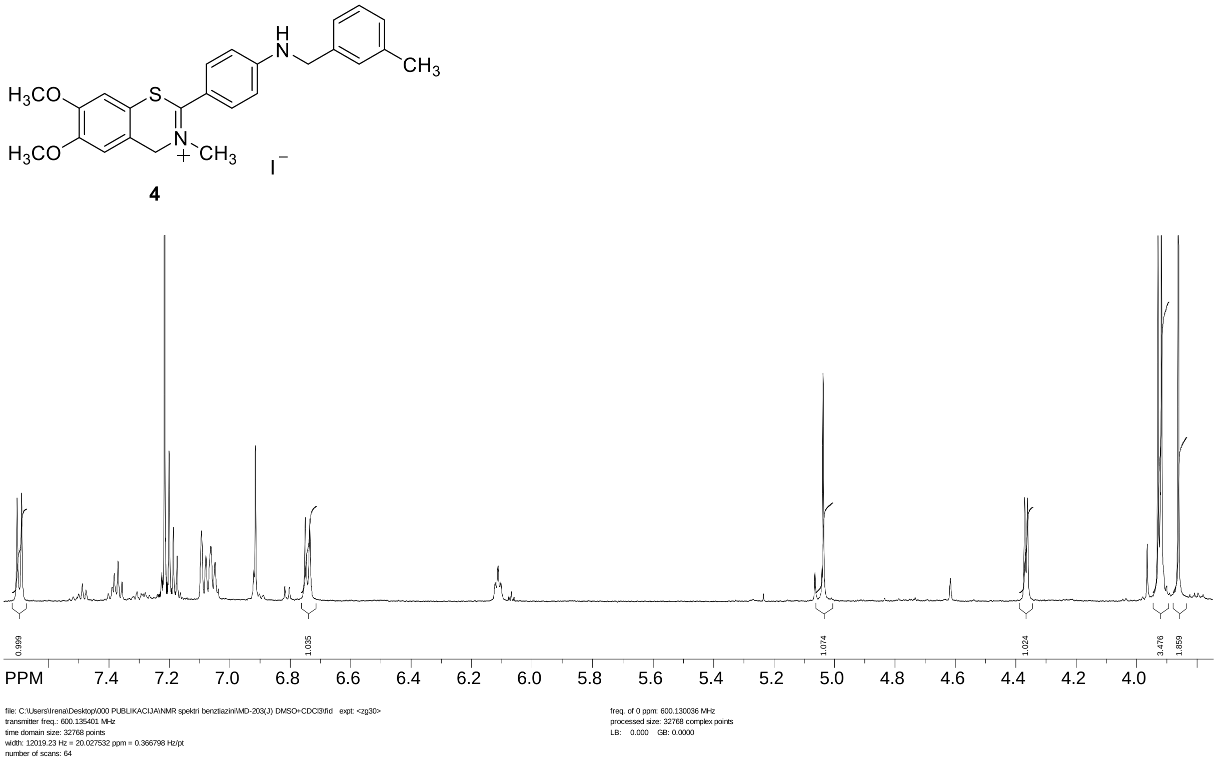

Figure S39. ^{1}H NMR ($CDCl_3$ + DMSO) spectrum of benzothiazine **4**.

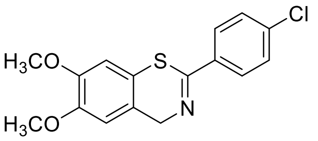

8'

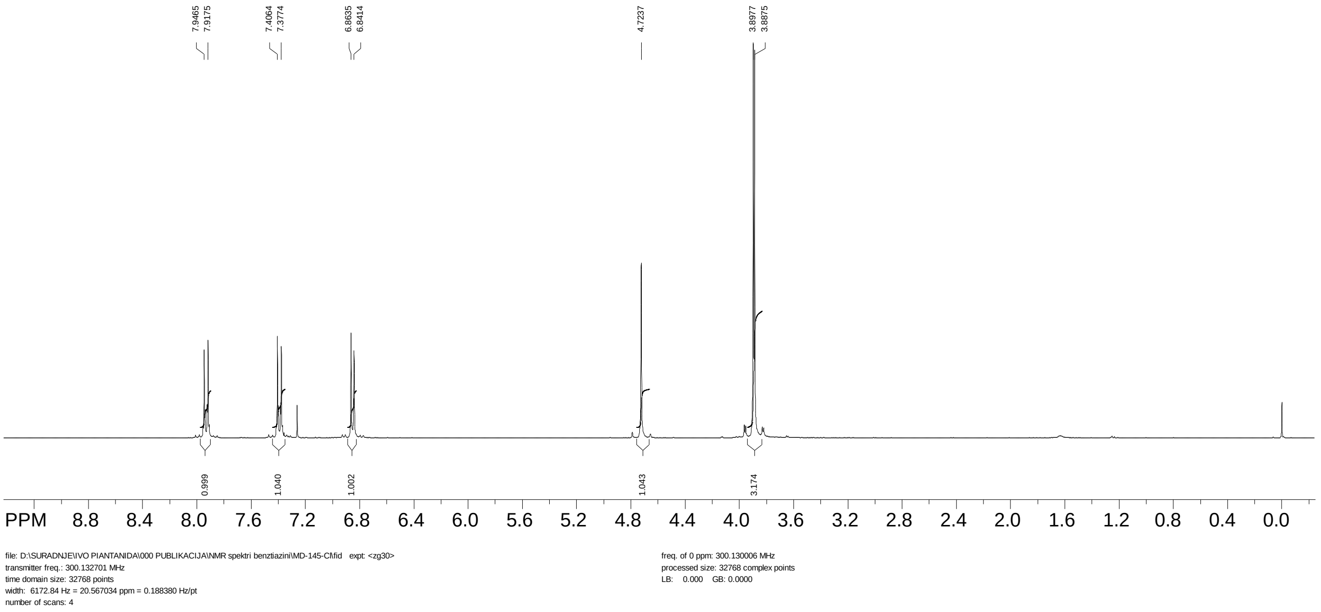

Figure S40. ^{1}H NMR ($CDCl_3$) spectrum of benzothiazine **8'**.

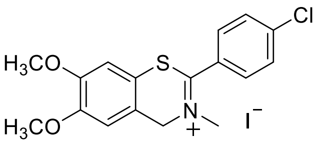

8

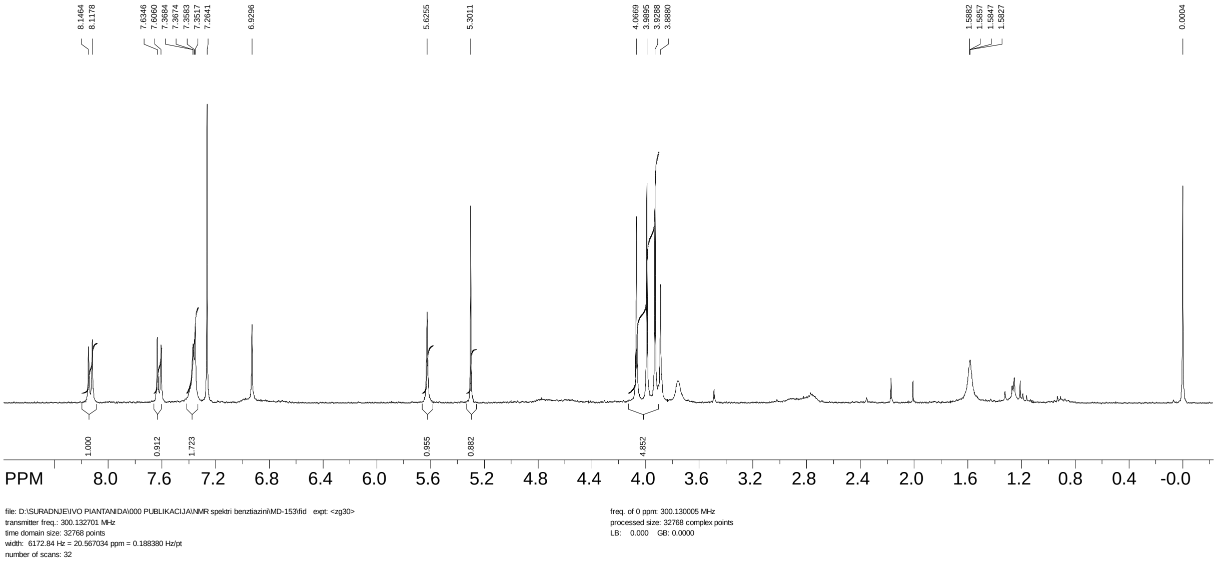

Figure S41. ^{1}H NMR ($CDCl_3$) spectrum of benzothiazine **8**.

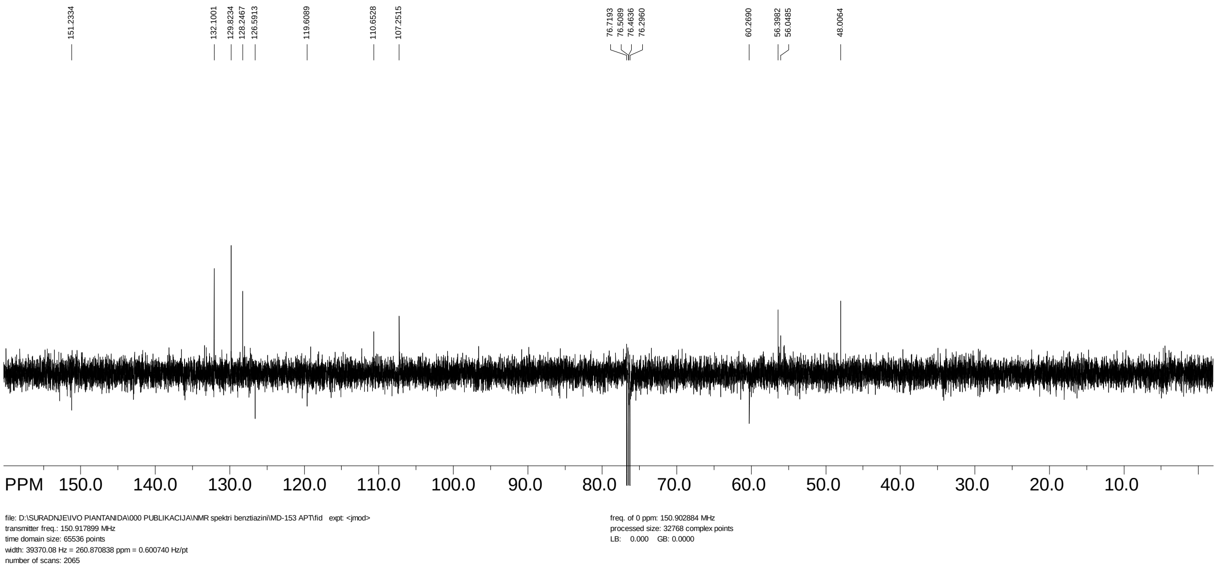

Figure S42. APT ($CDCl_3$) spectrum of benzothiazine **8**.

4. MS spectra and HRMS analyses of benzothiazines

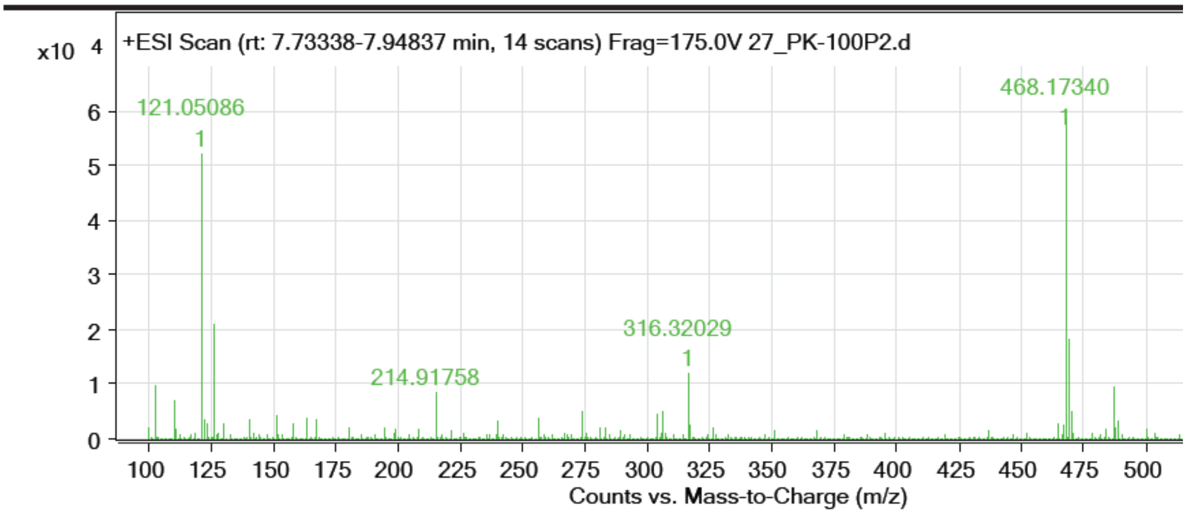

Formula Calculator Results

Formula	Best	Mass	Tgt Mass	Diff (ppm)	Ion Species	Score
C28 H25 N3 O2 S	True	467.16615	467.16675	1.29	C28 H26 N3 O2 S	97.59

Figure S43. MS spectrum and HRMS analysis of benzothiazine **1'**.

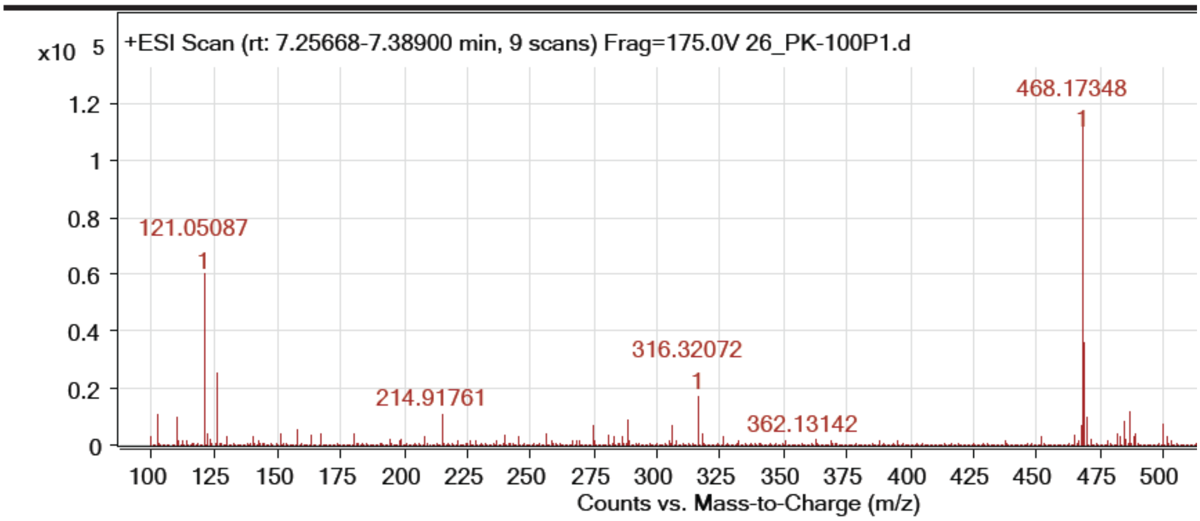

Formula Calculator Results

Formula	Best	Mass	Tgt Mass	Diff (ppm)	Ion Species	Score
C28 H25 N3 O2 S	True	467.16626	467.16675	1.05	C28 H26 N3 O2 S	98.64

Figure S44. MS spectrum and HRMS analysis of benzothiazine **2'**.

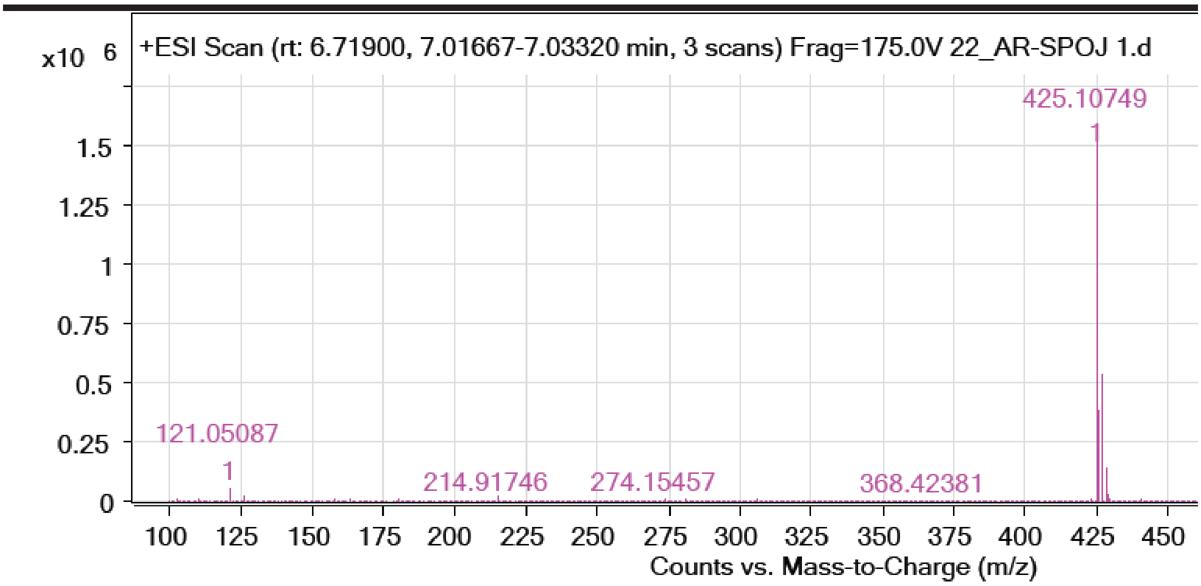

Formula Calculator Results

Formula	Best	Mass	Tgt Mass	Diff (ppm)	Ion Species	Score
C23 H21 Cl N2 O2 S	True	424.10042	424.10123	1.91	C23 H22 Cl N2 O2 S	96.17

Figure S45. MS spectrum and HRMS analysis of benzothiazine **3'**.

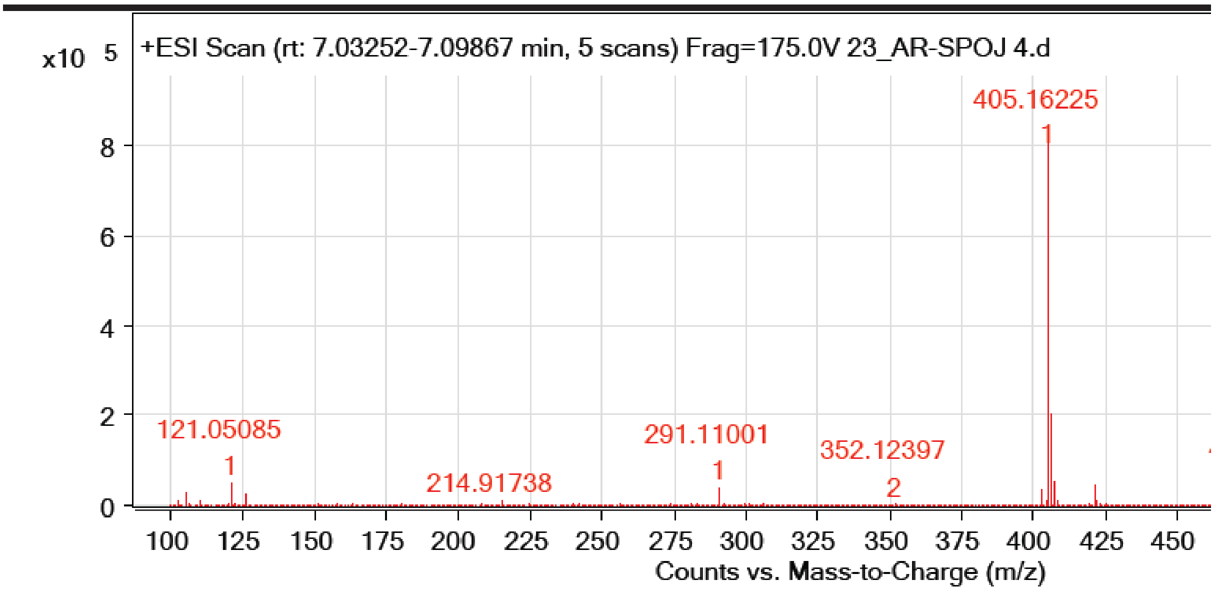

Formula Calculator Results

Formula	Best	Mass	Tgt Mass	Diff (ppm)	Ion Species	Score
C23 H22 N2 O S	True	374.1447	374.14528	1.55	C23 H23 N2 O S	88.81

Figure S46. MS spectrum and HRMS analysis of benzothiazine **4'**.

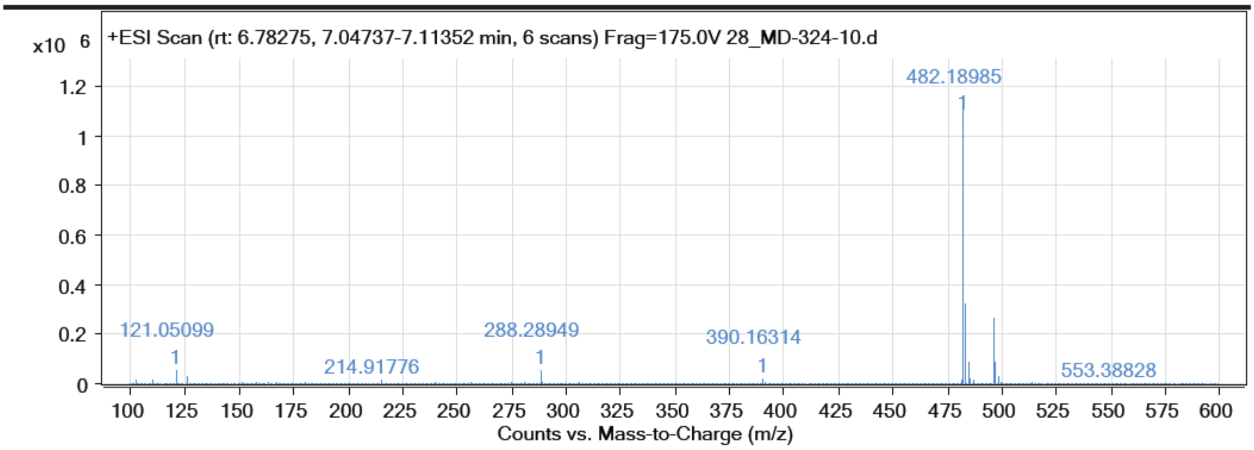

Formula Calculator Results

Formula	Best	Mass	Tgt Mass	Diff (ppm)	Ion Species	Score
C29 H27 N3 O2 S	True	481.18269	481.1824	-0.6	C29 H28 N3 O2 S	96.23

Figure S47. MS spectrum and HRMS analysis of benzothiazine **1**.

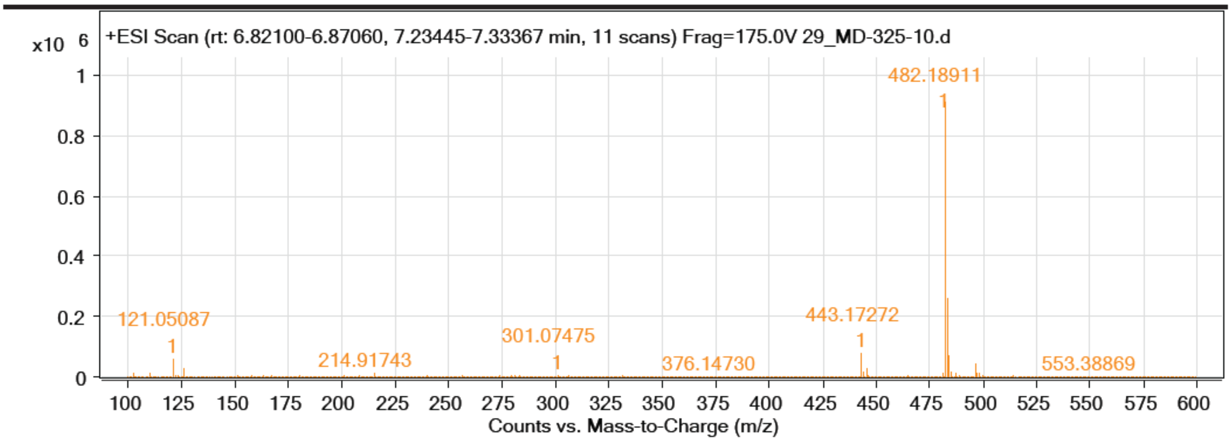

Formula Calculator Results

Formula	Best	Mass	Tgt Mass	Diff (ppm)	Ion Species	Score
C29 H27 N3 O2 S	True	481.18198	481.1824	0.87	C29 H28 N3 O2 S	95.67

Figure S48. MS spectrum and HRMS analysis of benzothiazine **2**.

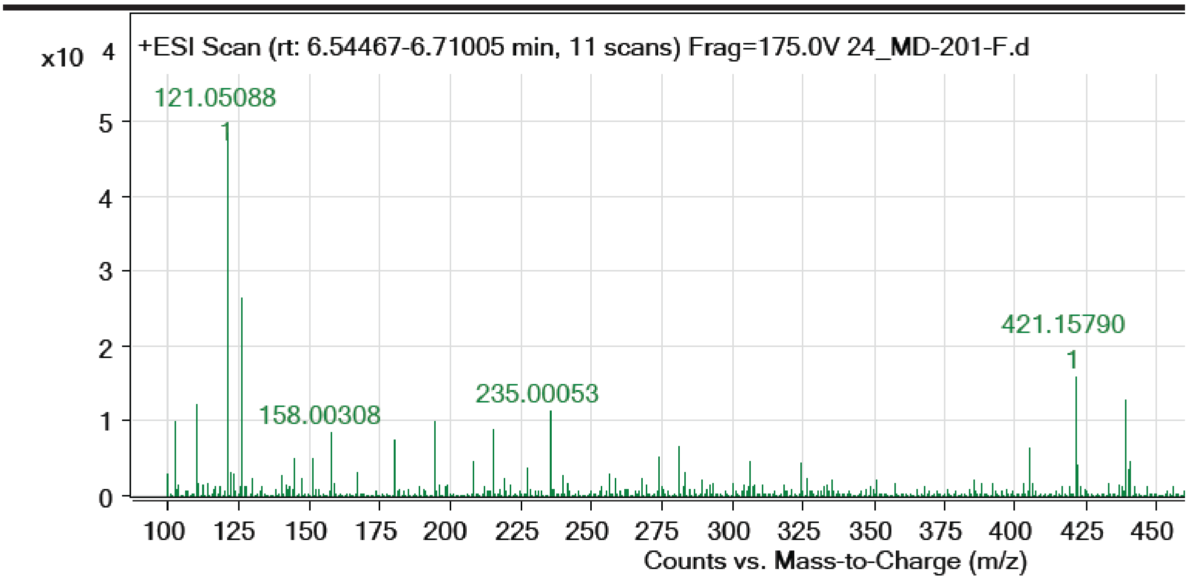

Formula Calculator Results

Formula	Best	Mass	Tgt Mass	Diff (ppm)	Ion Species	Score
C24 H23 Cl N2 O2 S	True	438.11649	438.11688	0.89	C24 H24 Cl N2 O2 S	98.33

Figure S49. MS spectrum and HRMS analysis of benzothiazine **3**.

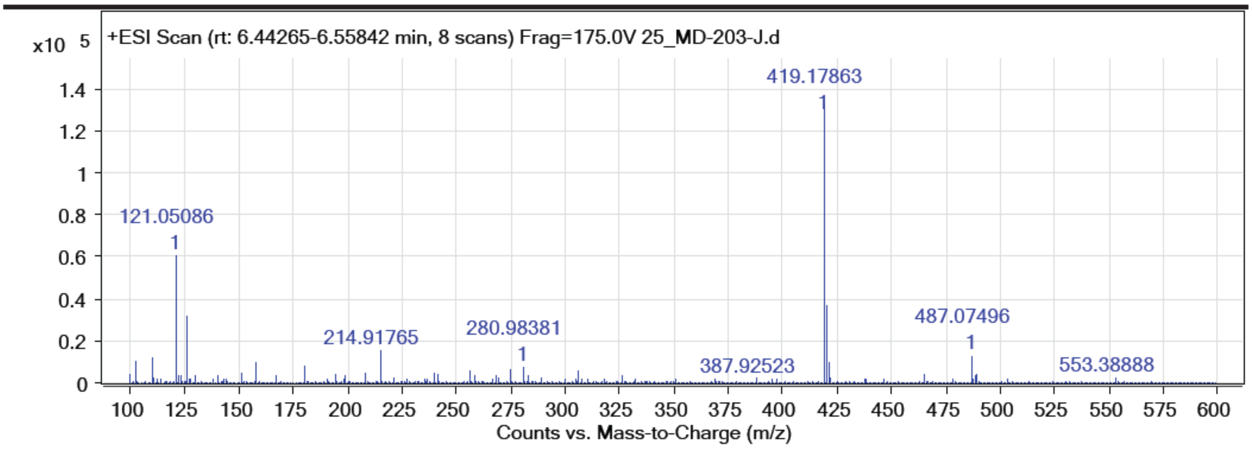

Formula Calculator Results

Formula	Best	Mass	Tgt Mass	Diff (ppm)	Ion Species	Score
C25 H26 N2 O2 S	True	418.17131	418.1715	0.46	C25 H27 N2 O2 S	98.41

Figure S50. MS spectrum and HRMS analysis of benzothiazine **4**.

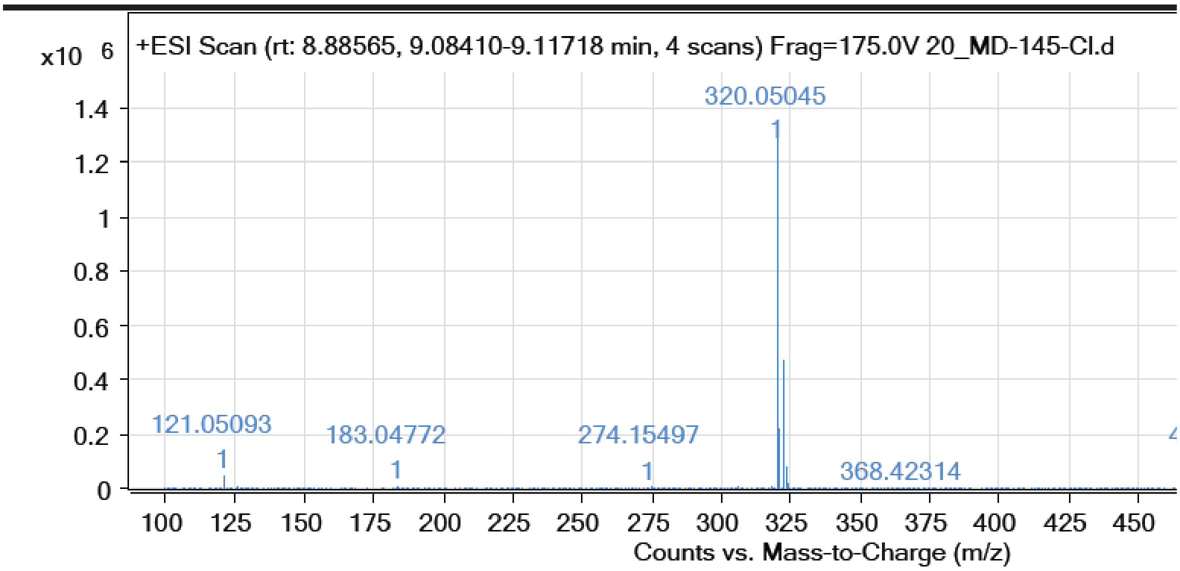

Formula Calculator Results

Formula	Best	Mass	Tgt Mass	Diff (ppm)	Ion Species	Score
C16 H14 Cl N O2 S	True	319.04324	319.04338	0.43	C16 H15 Cl N O2 S	99.11

Figure S51. MS spectrum and HRMS analysis of benzothiazine **8'**.

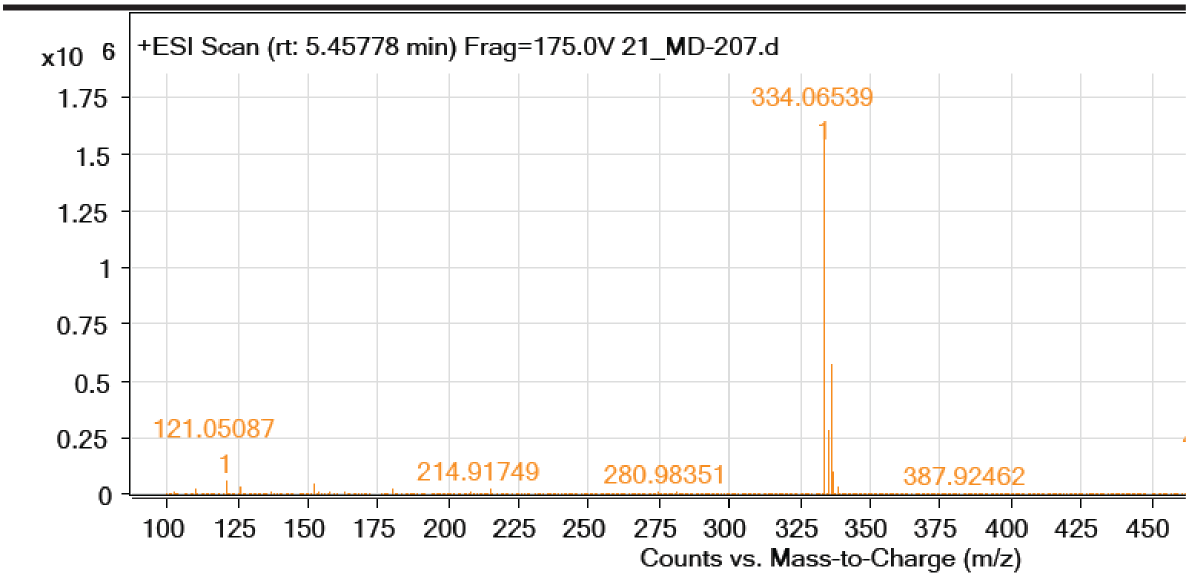

Formula Calculator Results

Formula	Best	Mass	Tgt Mass	Diff (ppm)	Ion Species	Score
C17 H16 Cl N O2 S	True	333.05817	333.05903	2.56	C17 H17 Cl N O2 S	96.7

Figure S52. MS spectrum and HRMS analysis of benzothiazine **8**.
